# The Novel J-Domain Protein Mrj1 Is Required for Mitochondrial Respiration and Virulence in Cryptococcus neoformans

**DOI:** 10.1128/mBio.01127-20

**Published:** 2020-06-09

**Authors:** Linda C. Horianopoulos, Guanggan Hu, Mélissa Caza, Kerstin Schmitt, Peter Overby, James D. Johnson, Oliver Valerius, Gerhard H. Braus, James W. Kronstad

**Affiliations:** a Michael Smith Laboratories, Department of Microbiology and Immunology, University of British Columbia, Vancouver, British Columbia, Canada; b Institut für Mikrobiologie & Genetik, Georg-August-Universität, Göttingen, Germany; c Department of Cellular and Physiological Sciences, University of British Columbia, Vancouver, British Columbia, Canada; Duke University

**Keywords:** fungal pathogenesis, alternative oxidase, capsule, cell wall, complex III, cryptococcosis, electron transport chain, mouse model, oxygen consumption

## Abstract

The opportunistic fungal pathogen Cryptococcus neoformans must adapt to the mammalian environment to establish an infection. Proteins facilitating adaptation to novel environments, such as chaperones, may be required for virulence. In this study, we identified a novel mitochondrial co-chaperone, Mrj1 (mitochondrial respiration J-domain protein 1), necessary for virulence in C. neoformans. The *mrj1Δ* and J-domain-inactivated mutants had general growth defects at both routine laboratory and human body temperatures and were deficient in the major virulence factor of capsule elaboration. The latter phenotype was associated with cell wall changes and increased capsular polysaccharide shedding. Accordingly, the *mrj1Δ* mutant was avirulent in a murine model of cryptococcosis. Mrj1 has a mitochondrial localization and co-immunoprecipitated with Qcr2, a core component of complex III of the electron transport chain. The *mrj1* mutants were deficient in mitochondrial functions, including growth on alternative carbon sources, growth without iron, and mitochondrial polarization. They were also insensitive to complex III inhibitors and hypersensitive to an alternative oxidase (AOX) inhibitor, suggesting that Mrj1 functions in respiration. In support of this conclusion, *mrj1* mutants also had elevated basal oxygen consumption rates which were completely abolished by the addition of the AOX inhibitor, confirming that Mrj1 is required for mitochondrial respiration through complexes III and IV. Furthermore, inhibition of complex III phenocopied the capsule and cell wall defects of the *mrj1* mutants. Taken together, these results indicate that Mrj1 is required for normal mitochondrial respiration, a key aspect of adaptation to the host environment and virulence.

## Abstract (precis)

Cryptococcus neoformans is the causative agent of cryptococcal meningitis, a disease responsible for ∼15% of all HIV-related deaths. Unfortunately, development of antifungal drugs is challenging because potential targets are conserved between humans and C. neoformans. In this context, we characterized a unique J-domain protein, Mrj1, which lacks orthologs in humans. We showed that Mrj1 was required for normal mitochondrial respiration and that mutants lacking Mrj1 were deficient in growth, capsule elaboration, and virulence. Furthermore, we were able to phenocopy the defects in growth and capsule elaboration by inhibiting respiration. This result suggests that the role of Mrj1 in mitochondrial function was responsible for the observed virulence defects and reinforces the importance of mitochondria to fungal pathogenesis. Mitochondria are difficult to target, as their function is also key to human cells; however, Mrj1 presents an opportunity to target a unique fungal protein required for mitochondrial function and virulence in C. neoformans.

## INTRODUCTION

As an opportunistic pathogen, the ability of Cryptococcus neoformans to adapt to conditions in mammalian hosts is essential for pathogenesis. At a basic level, adaptation includes evasion of the host immune system and survival at normal mammalian body temperature (1). Imperative to this adaptation is the ability to ensure that proper protein folding and complex assembly occur in conditions of stress. One class of proteins which potentially contribute to host adaptation are the molecular chaperones that help maintain proteostasis in response to changing environmental conditions (2–5). Several studies in C. neoformans have reported transcriptional control of molecular chaperones and heat shock proteins (HSPs) in response to increased temperature (6) and regulation occurring via transcription factors and signaling functions known to play roles in virulence, such as Rim101 and Pka1 (7–9). Proteomic analyses also identified HSPs in extracellular vesicles known to carry virulence-associated enzymes and capsule material, further supporting a role for chaperones in C. neoformans virulence beyond mitigation of heat-induced stress in the host (10).

Most studies conducted on HSPs in C. neoformans have focused on Hsp70s and Hsp90s, likely due to their abundance and crucial roles in multiple pathways. For example, the deletion of *SSA1*, encoding an Hsp70 protein, attenuates virulence in a mouse model of cryptococcosis due to reduction in both melanization and immunomodulation in the mutant compared to the wild-type (WT) strain (11, 12). Pharmacological inhibition of Hsp90 also attenuates virulence in a Caenorhabditis elegans model of cryptococcosis (13). Later studies also showed that Hsp90 was required for thermotolerance and localized to the cell surface (14). Surprisingly, the J-domain proteins (JDPs; often referred to as Hsp40s) that act as co-chaperones and critically direct Hsp70 function have not been characterized in C. neoformans despite the large size of the family and their reported importance in other fungal pathogens (15–19).

In general, JDPs have two major roles: (i) to recruit nonnative client proteins to Hsp70s and (ii) to activate the ATPase activity of Hsp70s necessary for tight binding with the client protein (20). There are several well-conserved JDPs that execute these general functions to aid in protein folding and protein complex assembly and to prevent protein aggregation. However, there are also several JDPs with specialized roles in Saccharomyces cerevisiae including disassembly of clathrin during endocytosis, biogenesis of iron-sulfur clusters, translocation of proteins across membranes, ribosome biogenesis, and pre-mRNA splicing (20, 21). It has been suggested that the roles of several JDPs in S. cerevisiae are heavily influenced by their spatial orientation and localization with an organelle (21, 22). For example, Jac1 participates in the specialized function of Fe-S cluster biogenesis in mitochondria (23). Several JDPs have been characterized in other fungal pathogens, including MHF16 and MHF21 which are required for conidiation in Magnaporthe oryzae (17), as well as Ydj1 which contributes to thermotolerance and phenotypic switching in Candida albicans (16). Furthermore, several of the JDPs are necessary for virulence through maintaining organellar function including Dnj1, which is required for endoplasmic reticulum homeostasis in Ustilago maydis (15), as well as Mas5 and Mdj1, which are important for tolerance to oxidative stress in Beauveria bassiana (perhaps reflecting their putative mitochondrial association) (18, 19).

In C. neoformans, information about the roles of JDPs is limited to differential expression in serial analysis of gene expression (SAGE), microarray, and transcriptome sequencing (RNA-Seq) experiments (6–9), as well as reduced infectivity of one mutant lacking a JDP gene in a pooled infection of signature tagged knockout mutants (24). Given the roles of JDPs in the pathogenesis of other fungi and their general roles in proteostasis (particularly under temperature stress), these co-chaperones are strong candidates to contribute to host adaptation and virulence in C. neoformans. In this study, we examined the JDP family and specifically characterized the role of Mrj1, a protein that is highly divergent in amino acid sequence from any other characterized JDPs. The *mrj1Δ* deletion mutant as well as a mutant with a single amino acid change in the J domain of Mrj1 have slow growth phenotypes and are temperature sensitive. A thorough characterization of these mutants revealed that Mrj1 has roles in mitochondrial function, capsule elaboration, thermotolerance, cell wall architecture, and virulence. This protein is localized to mitochondria and interacts with Qcr2, a core component of complex III of the electron transport chain (ETC). Furthermore, mitochondrial respiration was impaired in the mutants, specifically, the mutants were found to reduce oxygen exclusively at the alternative oxidase and be deficient in electron flow through complexes III and IV. Further studies using chemical inhibition of complex III to disrupt the ETC suggested that the phenotypes and virulence defects of *mrj1* mutants are driven by defective mitochondrial respiration.

## RESULTS

### Identification of J-domain proteins and characterization of Mrj1 in C. neoformans.

We initially identified and performed an *in silico* characterization of the JDP family in C. neoformans to begin an analysis of the roles of these proteins in host adaptation. Specifically, the J-domain-containing proteins encoded in the genome of C. neoformans var. *grubii* strain H99 (25) were identified by a BLASTp analysis with the pfam J-domain consensus sequence (pfam00226). A total of 24 genes encoding JDPs were identified in the genome (see Table S1 in the supplemental material). Orthologs of these proteins in S. cerevisiae and Schizosaccharomyces pombe were retrieved from the EuPathDB Ortholog groups through FungiDB (26), and the subcellular localizations of these proteins were predicted using WoLF PSORT (27). Interestingly, several JDPs lacked any orthologs in the model yeast species.

10.1128/mBio.01127-20.8 TABLE S1 The gene identifiers (IDs) of the J-domain proteins in C. neoformans. Download Table S1, PDF file, 0.02 MB. Copyright © 2020 Horianopoulos et al. 2020 Horianopoulos et al. This content is distributed under the terms of the Creative Commons Attribution 4.0 International license.

**FIG 1:**
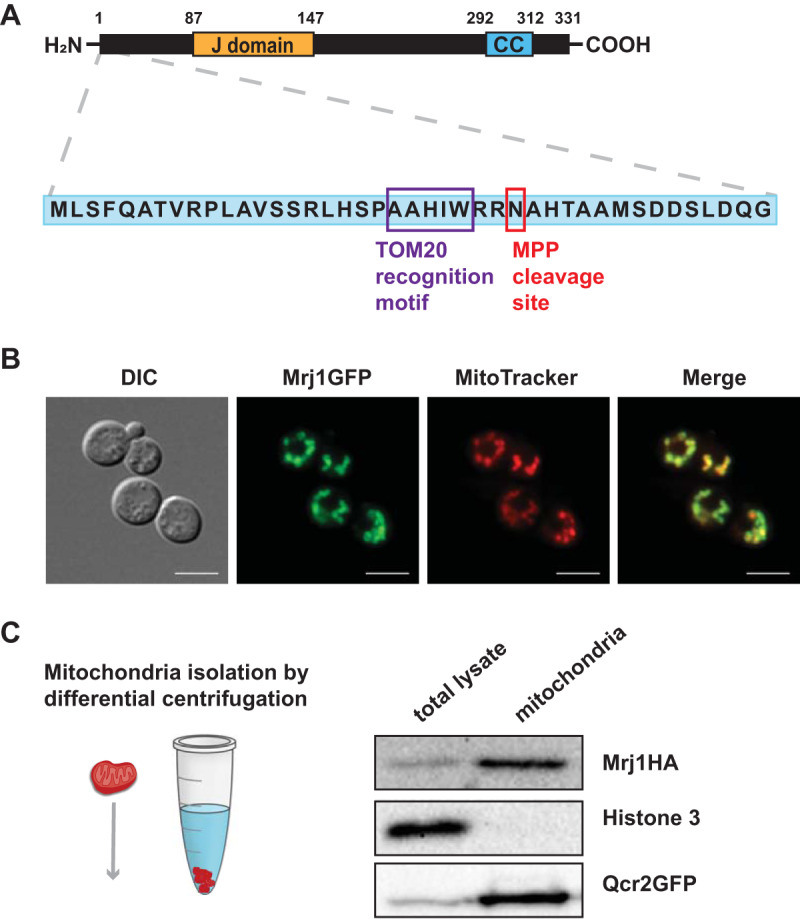
Mrj1 is a novel J-domain protein associated with mitochondria. (A) Diagram of Mrj1 to indicate specific features, including the separation of the J domain from the N terminus and the presence of a C-terminal coiled-coil (CC) domain. The amino acid positions of domains are indicated above the diagram. The protein was predicted to be mitochondrial based on the presence of a mitochondrial presequence and a mitochondrial processing peptidase (MPP) cleavage site. (B) Experimental confirmation of a mitochondrial association using a strain expressing a GFP-tagged version of Mrj1 and costaining with MitoTracker CMXRos. Bars = 5 μm. (C) Mrj1 was experimentally confirmed to be mitochondrial after immunoblotting for HA-tagged Mrj1 in both total cell lysate and in protein in mitochondria isolated by differential centrifugation. Qcr2GFP was used as a mitochondrial control, and histone 3 was used to confirm that mitochondrial fractions were free from nuclear proteins.

One of the JDPs, which we named Mrj1 (CNAG_00938), was of particular interest because it was divergent from JDPs in other species and had orthologs only in *Cryptococcus*, *Kwoniella*, and *Tremella* species. Mrj1 was predicted to be mitochondrial, and it is a type III JDP lacking an N-terminal region J domain (20, 22) (Fig. 1A). By comparison with the annotation of the *MRJ1* gene from the serotype D strain JEC21 (C. neoformans var. *neoformans*), we determined that the entry for CNAG_00938 was misannotated and that Mrj1 has an N-terminal region before the J domain; this region is absent from the annotation in NCBI (Fig. 1A). We experimentally confirmed this by amplification and sequencing of the full-length transcript from cDNA and by determining the size of the protein by immunoblot analysis (38 kDa). Outside of the J domain, there is a predicted coiled-coil region in Mrj1. Furthermore, there are no close orthologs in well-characterized fungi, with the most similar JDP in *U. maydis* sharing only 26% identity over 77% coverage (see Fig. S1 in the supplemental material). The unique features of Mrj1 based on sequence analysis prompted us to focus on this protein for further investigation.

10.1128/mBio.01127-20.1 FIG S1 The amino acid sequence of Mrj1 is divergent from other J-domain proteins outside the conserved J domain. The top BLASTp hits from Sporisorium reilianum, Ustilago maydis, and Ustilago hordei were aligned with Mrj1 from C. neoformans and C. gattii. The sequences are similar within the boxed J domain; however, Mrj1 from *Cryptococcus* spp. is largely distinct from the other amino acid sequences outside this highly conserved region. Download FIG S1, TIF file, 2.8 MB. Copyright © 2020 Horianopoulos et al. 2020 Horianopoulos et al. This content is distributed under the terms of the Creative Commons Attribution 4.0 International license.

### Mitochondrial localization of Mrj1.

The roles of several of the specialized JDPs in S. cerevisiae are influenced by their location within a specific organelle (21). We therefore examined the localization of Mrj1 in C. neoformans in light of the predicted mitochondrial presequence and the mitochondrial processing peptidase cleavage site predicted by MitoFates (Fig. 1A) (28). An Mrj1-GFP (green fluorescent protein) fusion protein was expressed from the elongation factor promoter at the safe haven locus in the genome (29). With this strain, Mrj1-GFP was observed to colocalize with mitochondria through costaining with MitoTracker CMXRos (Fig. 1B). Positive correlations of Pearson’s *R* values were found in all cells with a mean *R* value of 0.88 (0.05 standard deviation [SD], *n* = 15). We also showed that hemagglutinin (HA)-tagged Mrj1 (Mrj1-HA) was enriched in mitochondria isolated by differential centrifugation from C. neoformans (Fig. 1C). Both the Mrj1-GFP and Mrj1-HA fusion proteins complemented the growth defect of the *mrj1Δ* mutant (Fig. S2), thus demonstrating that these tagged versions of Mrj1 were functional. Overall, the results of our microscopy and immunoblotting analyses support the *in silico* prediction that Mrj1 is localized to the mitochondria.

10.1128/mBio.01127-20.2 FIG S2 Tagged versions of Mrj1 complemented the mutant growth defect, and expression of tagged Qcr2 did not influence growth. The growth curves in YNB indicate that tagging Mrj1 with either C-terminal HA (*mrj1Δ*::MRJ1HA) or GFP (*pEF1*-Mrj1GFP) complemented the growth back to the WT level. Furthermore, expression of Qcr2-GFP had no effect on the growth of either the WT, Mrj1-HA strain, or mutant. Error bars on all growth curves represent the standard deviations for three biological replicates. Download FIG S2, TIF file, 1.4 MB. Copyright © 2020 Horianopoulos et al. 2020 Horianopoulos et al. This content is distributed under the terms of the Creative Commons Attribution 4.0 International license.

### Expression of Mrj1 upon heat shock treatment.

**FIG 2:**
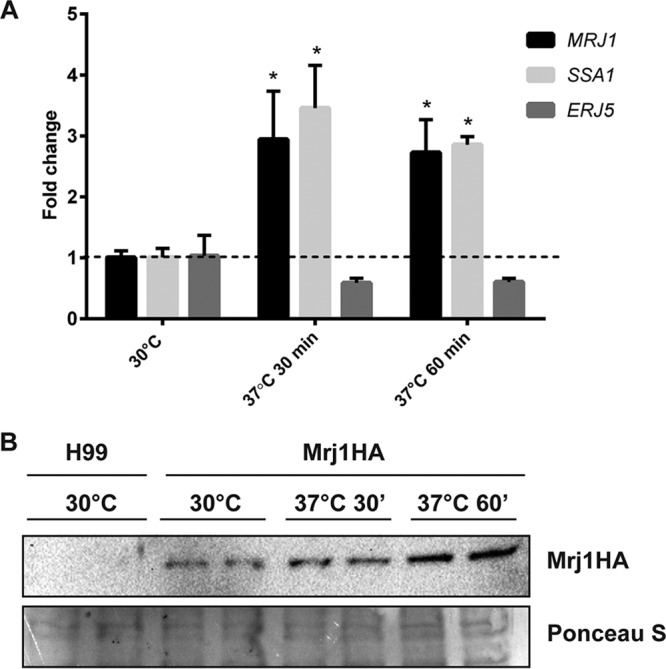
The expression of Mrj1 is elevated in response to incubation at 37°C. (A) The relative *MRJ1* expression levels were measured using RT-qPCR after incubating C. neoformans for 30 and 60 min at 37°C. The transcript levels for a known heat shock-responsive gene, the *SSA1* gene encoding an Hsp70 chaperone, and a gene for a UPR-responsive J-domain protein, *ERJ5*, are shown for comparison. Each bar represents the average and standard deviation for three biological replicates, and statistically significant differences relative to the transcript levels at 30°C were determined by an unpaired *t* test (*, *P* < 0.05). (B) Detection of Mrj1-HA protein by immunoblot analysis with an anti-HA antibody after incubation of cells for 30 min (30’) and 60 min (60’) at 37°C; Ponceau S staining is shown as a loading control for the 30 μg of total protein loaded in each lane.

Because Mrj1 is predicted to be a co-chaperone, we investigated whether *MRJ1* expression and Mrj1 protein abundance were influenced by temperature. At the transcript level, the expression of *MRJ1* upon a temperature shift from 30°C to human body temperature (37°C) was examined using relative quantification by reverse transcription-PCR (RT-qPCR). For comparison, the expression of *SSA1* (CNAG_01727), encoding a heat shock protein of the Hsp70 family known to be upregulated upon a temperature upshift in C. neoformans (6), and *ERJ5* (CNAG_05700), encoding a co-chaperone required for protein folding in the endoplasmic reticulum which is upregulated during the unfolded protein response (UPR) (30), were also determined. The transcript levels for both *MRJ1* and *SSA1* were elevated at both 30 and 60 min following a temperature upshift from 30°C to 37°C, whereas expression of *ERJ5* was not induced (Fig. 2A). Mrj1 protein abundance was also evaluated given that the *MRJ1* transcript was elevated at 37°C. The Mrj1-HA strain was grown to log phase at 30°C, transferred to prewarmed media, and incubated at 37°C. The change in abundance of Mrj1-HA was assessed by immunoblot analysis, and the tagged protein was found to increase in abundance after 30 min and to further accumulate after 60 min (Fig. 2B). Together, these results support the conclusion that Mrj1 is a temperature-responsive protein.

### Growth defects of *mrj1Δ* and *mrj1Δ*::MRJ1H111Q mutants.

**FIG 3:**
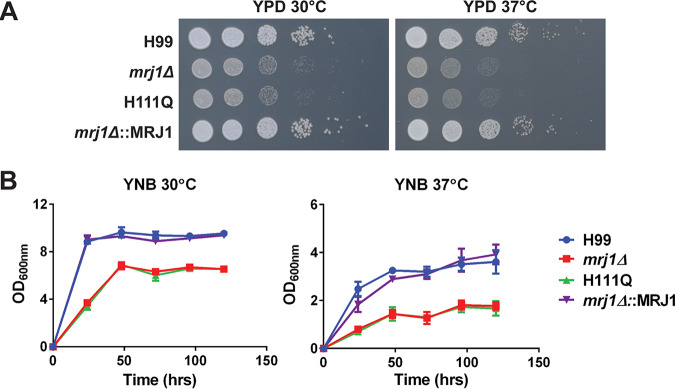
Mutants with defects in *MRJ1* are impaired for growth. (A) Spot assays on YPD plates revealed a growth defect for the *mrj1Δ* deletion mutant and the *mrj1Δ*::MRJ1H111Q (H111Q) site-directed mutant with abolished J-domain activity relative to the wild type (H99) or complemented (*mrj1Δ*::MRJ1) strains at 30°C and 37°C. (B) Liquid growth assays in YNB medium confirmed the growth defect in the mutants compared to the wild-type or complemented strains at 30°C and 37°C. Representative spot assays and growth curves are shown from three experiments. Time (in hours) is shown on the *x* axes. The error bars on the growth curves indicate standard deviations from three biological replicates.

To characterize the potential role of Mrj1 in the virulence of C. neoformans, a deletion mutant and a corresponding complemented strain were constructed using biolistic transformation and verified by PCR and Southern blot analysis (Fig. S3). The *MRJ1* knockout strain had impaired growth compared to the WT (H99) and complemented (*mrj1Δ*::MRJ1) strains, even under routine growth conditions on agar with rich medium (yeast extract-peptone-dextrose [YPD]) at 30°C (Fig. 3A). To ensure that this phenotype was due to the J-domain activity, a strain was generated with a single mutated codon to change the amino acid at position 111 (*mrj1Δ*::MRJ1H111Q). The mutation of the conserved histidine in the HPD motif between helices 2 and 3 of the J domain to glutamine (QPD) has previously been reported to abolish J-domain stimulation of Hsp70 ATPase as well as substrate release (31). The strain with this single amino acid change in Mrj1 was also found to have the same general growth defect on YPD at 30°C (Fig. 3A). The growth of the *mrj1Δ* and *mrj1Δ*::MRJ1H111Q mutants in liquid media was also impaired at 37°C in YNB (Fig. 3B and C). The slower growth of the mutants at 37°C is consistent with the expression data that Mrj1 is temperature responsive, but the growth defect under routine growth conditions suggests that the role of Mrj1 is not solely in mitigating temperature-induced stress.

10.1128/mBio.01127-20.3 FIG S3 Southern hybridization confirmation of the genotypes of the *mrj1Δ* and *mrj1Δ*::MRJ1 strains. DNA from the indicated strains was extracted and digested with BglII and BspEI at the indicated dashed lines, and genomic hybridization was performed using a ^32^P-labeled DNA probe (SP). The probe detected fragments of 3951 bp in the WT H99 strain, 4,726 bp in the deletion mutants, and 5,718 bp in the complemented strain. The weak bands above the observed fragments result from partial digestion. Download FIG S3, TIF file, 2.8 MB. Copyright © 2020 Horianopoulos et al. 2020 Horianopoulos et al. This content is distributed under the terms of the Creative Commons Attribution 4.0 International license.

### Capsule and cell wall defects of *mrj1* mutants.

**FIG 4:**
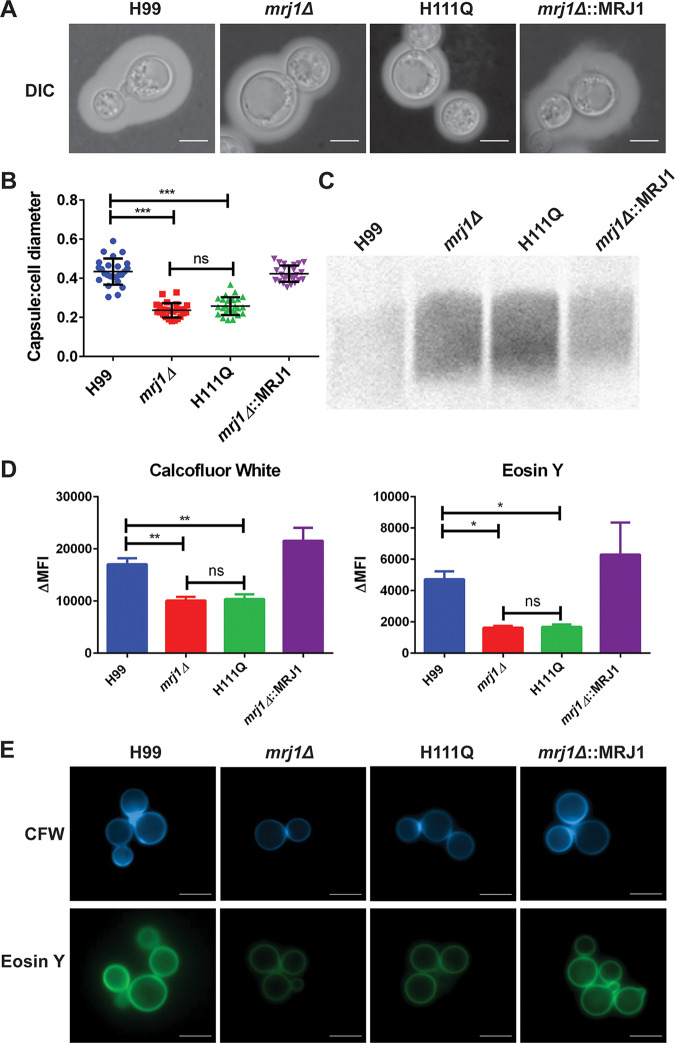
Mutants with defects in *MRJ1* are impaired in the attachment of capsule polysaccharide and have altered cell wall composition. (A and B) India ink staining to assess capsule size by microscopy after 48 h of growth in capsule-inducing medium (CIM) at 30°C. (A) The capsule sizes for cells of the *mrj1Δ* deletion mutant and *mrj1Δ*::MRJ1H111Q mutant (H111Q) were significantly smaller than for cells of the WT (H99) and the complemented strains (*mrj1Δ*::MRJ1). Representative images are shown. DIC, differential interference contrast. (B) Quantification of the ratio of capsule thickness to cell diameter for 50 cells per strain. (C) Blot of shed capsule polysaccharide detected with 18B7 antibody. The amount of shed capsule in culture supernatants after 48 h of growth in CIM was greater in the mutants than in the WT or complemented strains. (D) The cell wall architecture was found to be different with the mutant strains having less exposed chitin (calcofluor white [CFW]) and chitosan (eosin Y) than the WT or complemented strains as determined by flow cytometry. ΔMFI, change in mean fluorescence intensity. (E) Representative microscopy images of cell wall staining are shown (bars = 5 μm). Statistical significance determined by Mann-Whitney U tests is indicated by bars and asterisks as follows: * *P* < 0.05; **, *P* < 0.01; ***, *P* < 0.005; ns, not significant.

Generally, thermotolerance, capsule elaboration, and melanin synthesis are considered to be the major virulence factors of C. neoformans (1). The *mrj1* mutants displayed a considerable defect in capsule elaboration when capsule formation was induced using low-iron capsule-inducing medium (CIM). Both the *mrj1Δ* and *mrj1Δ*::MRJ1H111Q mutants had significantly lower capsule-to-cell body ratios compared to the WT and complemented strains (Fig. 4A and B). However, there was still a small amount of capsule present on the mutant cells, indicating that the biosynthesis of capsule was still occurring in the absence of Mrj1. Therefore, we examined the amount of shed capsular polysaccharide in the culture supernatant to determine whether the mutants had defects attaching capsule at the cell wall. After 48 h of capsule induction, a greater amount of shed capsule was found in the supernatant of the *mrj1Δ* and *mrj1Δ*::MRJ1H111Q mutants compared with the WT and complemented strains, suggesting that the mutant was impaired in capsule attachment rather than synthesis (Fig. 4C). We also employed cell wall staining of the mutants to determine whether an altered cell wall structure could potentially explain the defect in capsule attachment. We found that the *mrj1Δ* and *mrj1Δ*::MRJ1H111Q mutants had decreased exposed chitin and chitosan after staining the cells with calcofluor white or eosin Y and measuring the fluorescence using flow cytometry and fluorescence microscopy (Fig. 4D and E). Taken together, these data suggest that the cell wall structure is altered by loss of Mrj1 function, and this finding supports a model in which capsule attachment is impaired due to an altered cell surface.

### Virulence defect of an *mrj1Δ* mutant.

**FIG 5:**
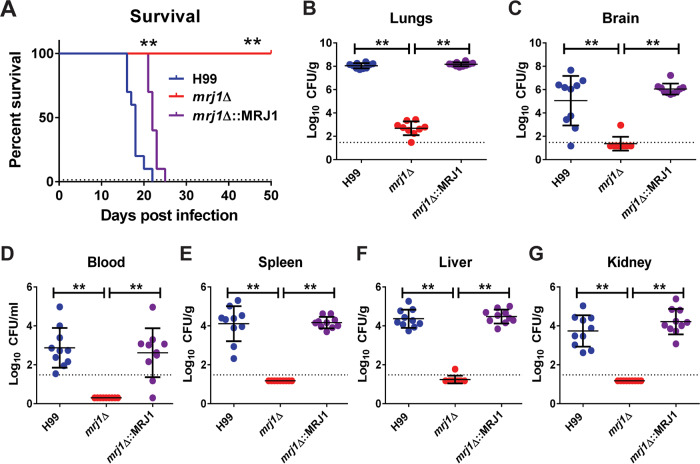
Mrj1 is important for the virulence of C. neoformans in a mouse model of cryptococcosis. (A) Mice infected with the mutant *(mrj1Δ*) survived to the end of a 50-day experiment, whereas mice inoculated with the WT (H99) or complemented strains *(mrj1Δ*::MRJ1) succumbed to infection between 16 and 25 days. Survival differences were determined using a log rank test (**, *P* < 0.01). (B to G) The fungal load for mice infected with the mutant was significantly lower than the fungal load for mice infected with the WT and complemented strains in the primary site of infection, the lung (B), as well as systemic organs, including the brain (C), blood (D), spleen (E), liver (F), and the kidney (G). Fungal burden was determined by measuring CFU, and differences between strains were evaluated by Mann-Whitney U tests (**, *P* < 0.01). The dotted lines indicate the CFU limit of detection.

Because an *mrj1Δ* mutant exhibited defects in two of the major virulence factors of C. neoformans, thermotolerance and capsule elaboration, we predicted that the mutant would be attenuated for virulence in an intranasal mouse model of cryptococcosis. This prediction was validated by the finding that all mice infected with the *mrj1Δ* mutant survived to the end of the experiment (50 days), whereas mice infected with the WT and complemented strains succumbed to the infection significantly earlier (days 16 to 25) (Fig. 5A). The organs collected when the mice succumbed to infection or, in the case of *mrj1Δ*, at the experimental endpoint, were homogenized and plated to quantitate the presence of viable cells. The numbers of CFU recovered from the lungs were significantly lower in the mice infected with the *mrj1Δ* mutant than in the mice infected with the WT and complemented strains (Fig. 5B). Importantly, the *mrj1Δ* mutant also showed a marked impairment in its ability to disseminate as indicated by its significantly lower abundance in the blood and other organs (liver, spleen, kidney, and brain) (Fig. 5C to G). Overall, the inability of the mutant to cause disease in mice and to disseminate beyond the lungs revealed that Mrj1 is an important contributor to the virulence of C. neoformans.

### Mitochondrial phenotypes of an *mrj1Δ* mutant.

**FIG 6:**
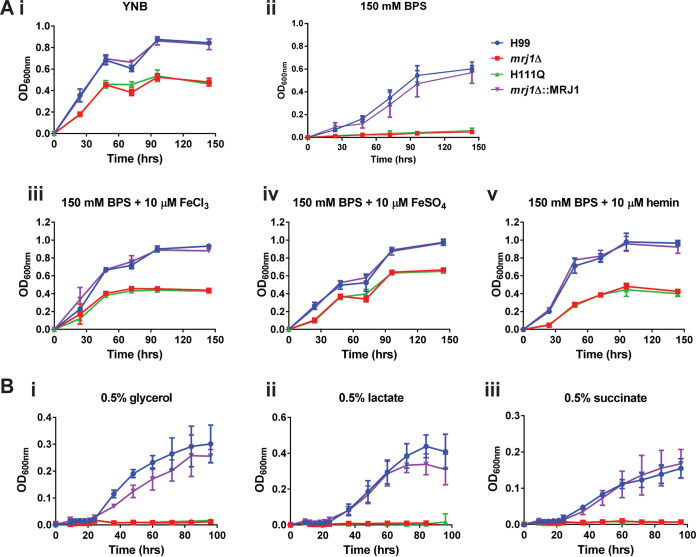
Mrj1 is required to support mitochondrial functions. (Ai) The *mrj1Δ* and H111Q mutants consistently have slower growth than the WT (H99) and complemented *(mrj1Δ*::MRJ1) strains. (Aii to v) Several conditions related to mitochondrial function were found to differentially affect mutant growth in comparison to standard growth conditions. Specifically, the mutants were incapable of growth in media with chelated iron (150 mM BPS) (ii) but were able to grow when iron in the form of ferric iron (10 μM FeCl_3_) (iii), ferrous iron (10 μM FeSO_4_) (iv), or hemin (10 μM) (v) was added back to the media. (B) The mutants were incapable of growing in YNB with glycerol, lactate, or succinate as alternative carbon sources (in place of glucose). Each growth curve is representative of at least two experiments, and in each experiment, the error bars represent the standard deviations for three biological replicates.

**FIG 7:**
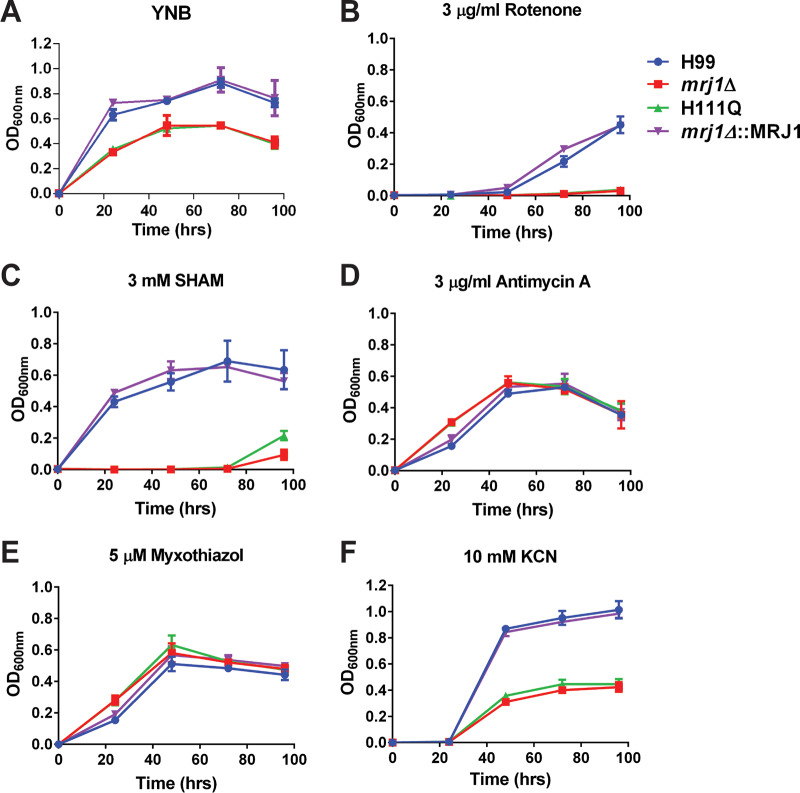
Inhibition of respiratory chain complexes differentially impacts strains lacking functional Mrj1. (A) The mutants (*mrj1Δ* and H111Q) normally do not grow as well as the WT (H99) and complemented (*mrj1Δ*::MRJ1) strains. (B and F) When grown in the presence of rotenone to inhibit complex I (B) or KCN to inhibit complex IV (F), all strains had reduced growth. (C) When grown in the presence of the alternative oxidase inhibitor, SHAM, the WT and complemented strains were unaffected, whereas the mutants were unable to grow. (D and E) Finally, the growth of mutants was unaffected by the complex III inhibitors antimycin A (D) as well as myxothiazol (E), whereas the growth of the WT and complement were decreased. Each growth curve is representative of three experiments, and the error bars represent the standard deviations of three biological replicates.

On the basis of the observed growth defects and mitochondrial localization of Mrj1, we next examined the growth of the mutants in several conditions selected to interrogate the function of Mrj1. Because mitochondria are important organelles for iron assimilation and utilization (32), we tested the *mrj1Δ* and *mrj1Δ*::MRJ1H111Q mutants for growth under low-iron conditions and in media with different iron sources. The mutants were unable to grow in the low-iron condition of yeast nitrogen base (YNB) supplemented with the iron chelator bathophenanthrolinedisulfonic acid (BPS) but were able to grow well regardless of the iron source added back to the media (Fig. 6A). This may suggest a defect in the iron labile pool and/or storage, as the addition of iron restored growth in the *mrj1* mutants. The growth of the mutants was also drastically impaired on the alternative carbon sources glycerol, lactate, or succinate that are metabolized via mitochondria-dependent processes (Fig. 6B). The susceptibility of the mutants to inhibitors of the electron transport chain (ETC) was also evaluated, yielding contrasting phenotypes upon inhibition of different respiratory complexes and providing evidence for the role of Mrj1 in mitochondrial function (Fig. 7). Inhibition of complex I with rotenone drastically decreased growth of all strains and completely abolished growth of *mrj1* mutants (Fig. 7B). When the alternative oxidase was inhibited using salicylhydroxymate (SHAM), the growth of the *mrj1Δ* and *mrj1Δ*::MRJ1H111Q mutants was dramatically reduced, although the WT and complemented strains showed little susceptibility (Fig. 7C). In contrast, the inhibitors of complex III decreased the growth of the WT and complemented strains to the level of the *mrj1Δ* and *mrj1Δ*::MRJ1H111Q mutants; however, they had no impact on the growth of the mutants themselves (Fig. 7D and E). Finally, inhibition of complex IV using KCN decreased the growth of all strains; however, due to the general growth defects of the *mrj1* mutants, it is difficult to say if they were differentially impacted (Fig. 7F). Although several mitochondrion-related phenotypes were observed in our growth assays, we note that other drugs targeting mitochondrial function did not differentially affect the growth of *mrj1* mutants; these drugs included tetracycline, chloramphenicol, diphenyleneiodonium, paraquat, and mitochondrial fission inhibitor 1 (mdivi-1) (Fig. S4). Overall, the growth phenotypes revealed by the experiments shown in Fig. 6 suggest that Mrj1 influences mitochondrial function through an impact on the ETC such that the alternative oxidase pathway becomes particularly important in the *mrj1* mutants. In this situation, we hypothesize that loss of Mrj1 causes dysregulation of complex III activity such that inhibition of the complex in the mutants has no impact on growth because electrons are flowing through the alternative oxidase to complete respiration.

10.1128/mBio.01127-20.4 FIG S4 Several mitochondrial targeting drugs do not differentially affect the growth of *mrj1* mutants. (A) The *mrj1Δ* knockout and J-domain-inactivated *mrj1Δ*::MRJ1H111Q (H111Q) strains had consistently reduced growth compared to the wild type (H99) and complemented strains (*mrj1Δ*::MRJ1). (B to F) These strains were challenged with several drugs that inhibit mitochondrial targets: the mitochondrial fission inhibitor mdivi-1 (B), the nitric oxide synthetase and complex I inhibitor diphenyleneiodonium (DPI) (C), the mitochondrial translation inhibitor chloramphenicol or tetracycline (D and E), and the superoxide generator paraquat (F). No clear differential impact on the mutants was noted when they were challenged with these drugs. Error bars represent the standard deviations for three biological replicates. Download FIG S4, TIF file, 1.3 MB. Copyright © 2020 Horianopoulos et al. 2020 Horianopoulos et al. This content is distributed under the terms of the Creative Commons Attribution 4.0 International license.

### Contribution of Mrj1 to mitochondrial membrane polarization.

**FIG 8:**
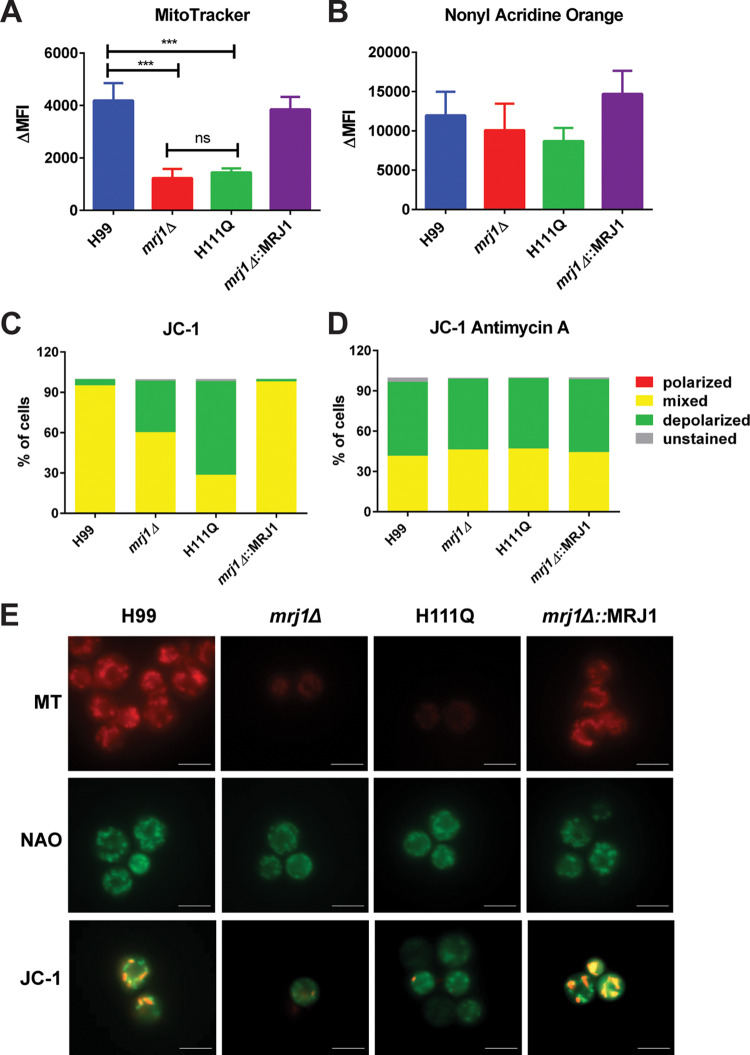
Mrj1 influences mitochondrial membrane polarization. (A and B) Changes in mean fluorescence intensities (ΔMFI) of different mitochondrial dyes were measured using flow cytometry to assess the impact of Mrj1 on mitochondrial function. (A) Polarized mitochondria were stained using the membrane potential-dependent dye MitoTracker CMXRos (MT), and significantly less fluorescence was observed in the mutants (*mrj1Δ* and H111Q) compared to the WT (H99) and complemented (*mrj1Δ*::MRJ1) strains. (B) Total mitochondria were stained with the membrane potential-independent dye nonyl acridine orange (NAO), and no significant differences were observed between the strains. In panels A and B, all bars represent the means and standard deviations of three biological replicates. Statistical significance was determined using one-way ANOVA with Dunn’s multiple comparisons (***, *P* < 0.005). (C) The dye JC-1 was used to determine the proportion of polarized mitochondria. The WT and complemented strains had a mixed population of mitochondria, whereas the mutants had an increased proportion of depolarized mitochondria. (D) All of the strains had a large proportion of cells with depolarized mitochondria when cells were grown in the presence of the complex III inhibitor antimycin A. (E) Representative microscopy images of mitochondrial staining are shown. Bars = 5 μm.

Because complex III, which is also known as the cytochrome *bc*_1_ complex, is involved in generating proton motive force through the Q cycle (33), we next employed flow cytometry to evaluate mitochondrial membrane polarization in the *mrj1* mutants. When cells were stained with the membrane potential-dependent dye, MitoTracker CMXRos, the *mrj1Δ* and *mrj1Δ*::MRJ1H111Q mutants had significantly less fluorescence compared to the WT and complemented strains (Fig. 8A). To ensure that this reduced fluorescence was due to decreased membrane polarization and not to a reduction in total mitochondria, cells were also stained with a membrane potential-independent dye, nonyl acridine orange, and no significant differences were observed between strains (Fig. 8B). Finally, staining with the dye JC-1, which forms aggregates and fluoresces red in polarized mitochondria, revealed that the mutants had a higher proportion of cells with depolarized mitochondria, whereas most cells in the WT and complemented strains had a mixture of polarized and depolarized mitochondria (Fig. 8C). To test whether this result was due to a lack of proton motive force at complex III in the mutant strains, we grew the wild type, mutants, and complemented strains in the presence of antimycin A and repeated the JC-1 staining. In this case, we found that inhibition of complex III increased the proportion of depolarized mitochondria in the wild-type and complemented strains to levels similar to those seen in the mutants (Fig. 8D). Furthermore, inhibition of the ETC using the other inhibitors, rotenone, SHAM, and myxothiazol, also decreased membrane polarization, reinforcing the idea that the ETC is important for maintaining membrane polarization (Fig. S5). The decreased staining of the *mrj1Δ* and *mrj1Δ*::MRJ1H111Q mutants with MitoTracker and the ability of antimycin A to phenocopy the decreased proportion of polarized mitochondria observed in the mutants using JC-1 support the conclusion that Mrj1 influences mitochondrial function and the level of the ETC.

10.1128/mBio.01127-20.5 FIG S5 Treatment with electron transport chain (ETC) inhibitors increased the proportion of cells with depolarized mitochondria. Wild-type cells were grown in the presence of ETC inhibitors, and an increased proportion of cells with depolarized mitochondria was observed by flow cytometry after JC-1 staining. Cells grown in the presence of KCN are not shown because insufficient growth was obtained to allow counting by flow cytometry. Bars represent the averages for three biological replicates. Download FIG S5, TIF file, 0.1 MB. Copyright © 2020 Horianopoulos et al. 2020 Horianopoulos et al. This content is distributed under the terms of the Creative Commons Attribution 4.0 International license.

### Interaction of Mrj1 with the complex III core protein Qcr2.

**FIG 9:**
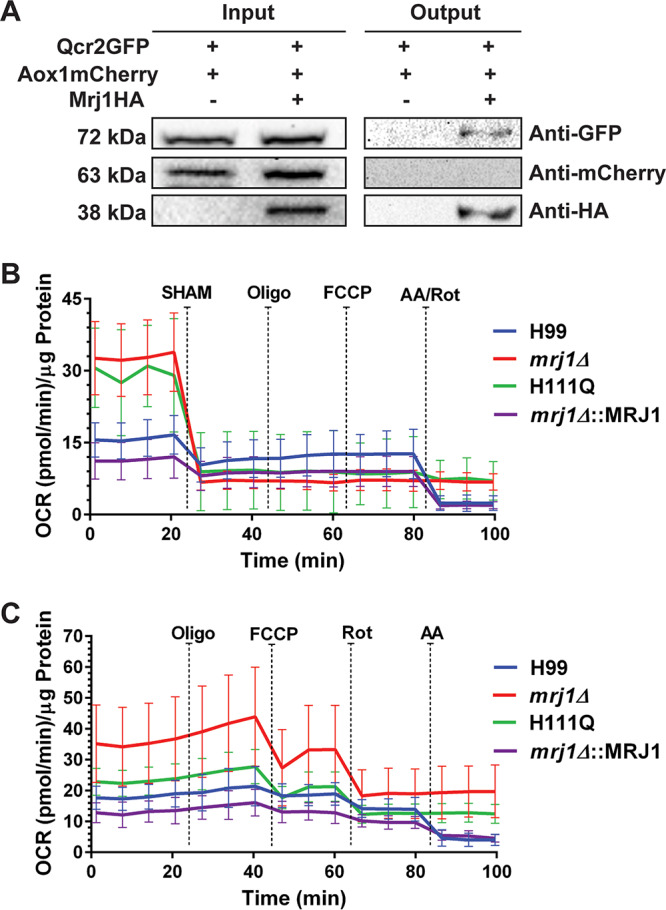
Mrj1 interacts with the ubiquinol cytochrome *c* reductase subunit Qcr2 and impacts mitochondrial respiration. (A) Immunoblot analysis of the protein lysate (Input) obtained after protein extraction from a strain expressing an N-terminal GFP-tagged Qcr2 and mCherry-tagged Aox1 (Qcr2-GFP:Aox1-mCherry) proteins and a strain expressing Qcr2-GFP, Aox1-mCherry, and N-terminal HA-tagged Mrj1 (Mrj1-HA:Qcr2-GFP:Aox1-mCherry) proteins showed that both Qcr2-GFP and Aox1-mCherry were expressed in both strains and Mrj1HA was expressed only in one strain. After co-immunoprecipitation with anti-HA magnetic beads, an immunoblot revealed an interaction between Mrj1 and Qcr2. That is, the Qcr2-GFP protein was only observed in the eluate (Output) in the strain expressing Mrj1-HA. Qcr2 was present in the eluate of the co-immunoprecipitation in all six repeats conducted. Aox1-mCherry was not detected in the eluate in either strain highlighting some level of specificity. (B and C) The oxygen consumption rates (OCR) of the wild-type (H99), *mrj1Δ*, *mrj1Δ*::MRJ1H111Q (H111Q), and *mrj1Δ*::MRJ1 strains measured using a Seahorse XFe96 analyzer with the indicated drugs sequentially injected at the time points indicated by dashed lines. The final concentrations of drugs used were 5 mM SHAM, 10 μM oligomycin (Oligo), 4 μM carbonyl-cyanide-4-(trifluoromethoxy) phenyhydrazone (FCCP), 4 μM rotenone (Rot), and 4 μM antimycin A (AA). The error bars indicate the standard deviations for eight biological replicates.

To further understand the role of Mrj1 in mitochondria and the impact of the protein on ETC, we identified candidate interacting partners of HA-tagged Mrj1 using affinity purification and mass spectrometry (AP-MS). A total of 192 proteins were identified (after filtering out contaminants, reverse peptides, and filtering for proteins that appeared in a minimum of two samples in each group, WT and Mrj1-HA). We focused on the proteins enriched in the eluate of the Mrj1-HA-tagged strain. This included several mitochondrial proteins involved in oxidative phosphorylation and metabolism (mitochondrial proteins highlighted in Table S2). Of these proteins, Qcr2 (CNAG_05179) is a subunit of ubiquinol cytochrome *c* reductase (complex III), and this protein was of particular interest because of our phenotypic observations with inhibitors of complex III. We therefore constructed strains containing Qcr2-GFP and Aox1-mCherry fusion proteins in both the WT background and the Mrj1-HA strain background to investigate the potential interaction of Qcr2 and Mrj1. The Aox1-mCherry fusion protein was used as a control to exclude the possibility that Mrj1 is nonspecifically interacting with mitochondrial proteins. When the lysates from these strains were incubated with anti-HA magnetic beads for co-immunoprecipitation, only Qcr2-GFP was found in the eluate of the Mrj1-HA strain, and no tagged proteins were in the eluate of the WT strain lacking the bait (Fig. 9A). This confirmation of the interaction between Mrj1 and Qcr2 provides further support that Mrj1 functions at the level of the ETC with some specificity for complex III in C. neoformans. Given that the interactions of J-domain proteins are often transient (34, 35), we cannot exclude the possibility that Mrj1 interacts with other proteins in mitochondria. However, the inability to detect Aox1-mCherry in the eluate suggests that Mrj1 is not promiscuously binding mitochondrial proteins.

10.1128/mBio.01127-20.9 TABLE S2 Proteins detected through AP-MS. Download Table S2, PDF file, 0.1 MB. Copyright © 2020 Horianopoulos et al. 2020 Horianopoulos et al. This content is distributed under the terms of the Creative Commons Attribution 4.0 International license.

### Characterization of mitochondrial respiration in *mrj1*Δ mutants.

Given the evidence that Mrj1 impacted the ETC, we measured the oxygen consumption rate (OCR) of the WT, *mrj1Δ*, *mrj1Δ*::MRJ1H111Q, and *mrj1Δ*::MRJ1 strains in YNB using a Seahorse XFe96 analyzer. Interestingly, the *mrj1* mutants had higher basal OCRs than the WT or complemented strains (Fig. 9B). However, when SHAM was used to inhibit the AOX, the OCR decreased dramatically in the mutants, and it did not decrease further after the addition of complex I/III inhibitors. In contrast, the OCR decreased after the addition of both SHAM and the complex I/III inhibitors for the wild-type and complemented strains. Furthermore, in the absence of SHAM, the OCR of the *mrj1* mutants was decreased by the complex I inhibitor (rotenone), but not by the complex III inhibitor (antimycin A), whereas the OCR in the wild-type and complemented strains decreased after addition of both inhibitors (Fig. 9C). Together, these results indicate that oxygen reduction is occurring exclusively at the AOX in the mutants, as SHAM completely abolishes OCR in these strains, and in its absence, only inhibition of complex I, which is upstream of AOX activity, decreases OCR. These data also suggest that there is less electron flow through complexes III and IV in the mutants. This is consistent with lower mitochondrial ROS, as measured using the MitoSOX Red Superoxide indicator, which is usually generated during electron flow through complex III (Fig. S6). We should also note that treatment with oligomycin to inhibit ATP-linked respiration had no effect on C. neoformans, as previously reported (36). FCCP [carbonyl-cyanide-4-(trifluoromethoxy)phenyhydrazone] was also used to uncouple proton motive force from oxygen consumption; however, we did not see an effect at any concentration tested during optimization of the OCR assays (1 μM, 2 μM, and 4 μM). Overall, our data on respiration in the *mrj1* mutants strongly agree with the mitochondrial defects indicated by the growth phenotypes and the decreased mitochondrial polarization and suggest that the interaction with Qcr2 is indicative of a functional role for Mrj1 in supporting mitochondrial respiration.

10.1128/mBio.01127-20.6 FIG S6 Mitochondrial superoxide formation is decreased in *mrj1* mutants. The *mrj1* mutants (*mrj1*Δ and H111Q) had decreased mean fluorescence intensity (ΔMFI) observed through flow cytometry compared to the wild-type (H99) and complemented (*mrj1*Δ::MRJ1) strains after staining with MitoSOX Red Superoxide indicator in YNB. Bars represent the averages for four biological replicates, and error bars indicate standard deviations. Download FIG S6, TIF file, 0.1 MB. Copyright © 2020 Horianopoulos et al. 2020 Horianopoulos et al. This content is distributed under the terms of the Creative Commons Attribution 4.0 International license.

### Importance of complex III and mitochondrial respiration to capsule and cell wall production.

**FIG 10:**
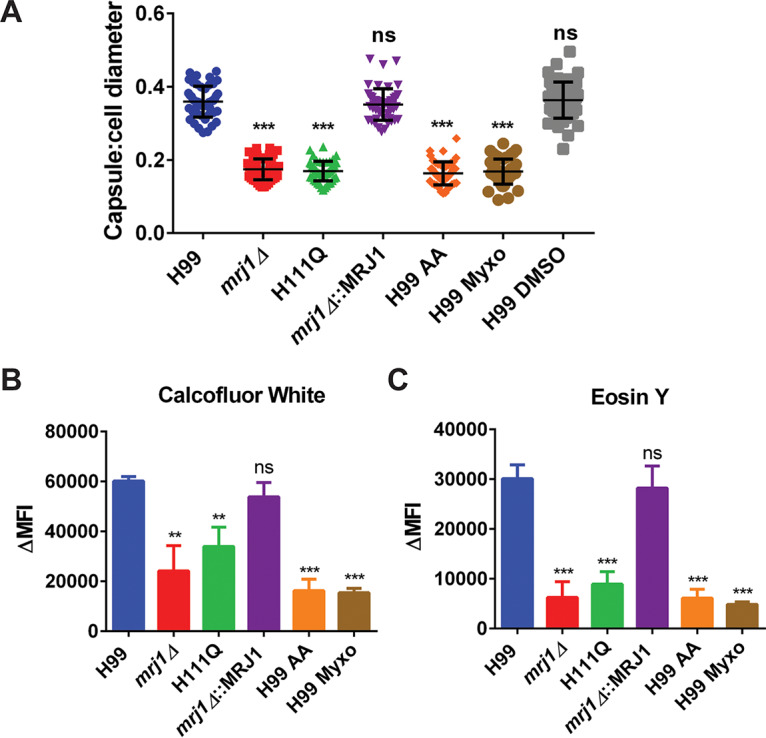
The capsule and cell wall changes observed in the absence of Mrj1 are phenocopied by treatment with complex III inhibitors. (A) After growth for 48 h at 30°C in capsule-inducing medium (CIM) in the presence of 3 μg/ml antimycin A (AA) or 5 μM myxothiazol (Myxo), the ratio of capsule thickness to cell body diameter of the WT (H99) cells was significantly smaller than untreated or vehicle control (dimethyl sulfoxide [DMSO])-treated cells of the WT and complemented strains. Notably, the capsule sizes of the treated WT cells were comparable those of the untreated mutant cells (*mrj1Δ* and H111Q). For each group, the capsule and cell body were measured for 50 cells. (B and C) The differences in cell wall staining measured by flow cytometry for the mutants were also phenocopied by treatment of WT with antimycin A or myxothiazol. Specifically, the mutants and the drug-treated WT cells had less exposed chitin (calcofluor white) and chitosan (eosin Y) than the untreated WT or complemented strains. Note that the control data presented here for the untreated strains were collected in an independent experiment from the one presented in Fig. 4. For both capsule and cell wall staining, error bars represent the standard deviations (of 50 cells for capsule, and three biological replicates for cell wall staining), and significant differences compared to the WT were determined by Mann-Whitney U tests (**, *P* < 0.01; ***, *P* < 0.005).

To ensure that the defects in virulence factor elaboration, in particular the capsule defect that we observed, were related to the proposed role of Mrj1 in influencing mitochondrial respiration, we examined the phenotypes of WT cells treated with the complex III inhibitors antimycin A and myxothiazol. When cells were grown in capsule-inducing medium in the presence of these drugs, the cells displayed similar phenotypes in terms of capsule size as the *mrj1* mutants (Fig. 10A). The cell wall architecture was also interrogated after growth in the presence of the complex III inhibitors. These inhibitors caused similar phenotypes, as loss of Mrj1 in the mutants in terms of reduced chitin and chitosan staining in the cell wall (Fig. 10B and C). Importantly, these results highlight a major role for mitochondrial function in influencing cell wall architecture and capsule attachment at the cell surface. Furthermore, these data suggest that the impact of Mrj1 on complex III function is sufficient to explain the capsule and cell wall defects observed in the mutants. It should be noted that inhibition of complex III and other ETC complexes has previously been reported to reduce capsule size in C. neoformans (37). Similarly, we also found that capsule size is decreased upon inhibition of complexes I and IV (Fig. S7). These findings reinforce the importance of the ETC in virulence factor elaboration in C. neoformans and further highlight how disruption of mitochondrial respiration in *mrj1* mutants impacted capsule elaboration and ultimately virulence.

10.1128/mBio.01127-20.7 FIG S7 Electron transport chain (ETC) inhibitors decreased the ratio of capsule size to cell diameter. Capsule size was reduced when capsule production was induced in wild-type cells grown in the presence of ETC inhibitors. There was no significant differences in the capsule size produced by *mrj1Δ* and the wild-type cells treated with complex III inhibitors (antimycin A and myxothiazol). In contrast, capsules were modestly but significantly larger in cells treated with the complex I inhibitor rotenone and significantly smaller in cells treated with the complex IV inhibitor KCN. The statistical significance of these differences compared to the *mrj1Δ* mutant was determined by a one-way ANOVA with Dunn’s multiple-comparison test (ns, not significant; *, *P* < 0.05; ***, *P* < 0.005). Download FIG S7, TIF file, 0.1 MB. Copyright © 2020 Horianopoulos et al. 2020 Horianopoulos et al. This content is distributed under the terms of the Creative Commons Attribution 4.0 International license.

## DISCUSSION

Our findings indicate that Mrj1 is a divergent JDP that contributes to the virulence of C. neoformans by supporting mitochondrial respiration. Mrj1 colocalizes with mitochondria, thus prompting a thorough characterization of its role in this organelle and the discovery that *mrj1* mutants differed from the wild type in key aspects of mitochondrial function. In particular, the mutants were unable to grow on alternative carbon sources and in low-iron media, and they displayed intriguing phenotypes when challenged with ETC inhibitors. While they were hypersensitive to an inhibitor of the alternative oxidase, they were insensitive to two complex III inhibitors. The mutants also had a decreased proportion of polarized mitochondria per cell. The decrease in polarized mitochondria was attributed to altered ETC activity based on the mutants’ lack of susceptibility to complex III inhibitors. Furthermore, a complex III inhibitor phenocopied the mutants’ decreased proportion of polarized cells. A core component of complex III, Qcr2, interacted with Mrj1 based on an AP-MS experiment, and this interaction was confirmed by co-immunoprecipitation using HA-tagged Mrj1 and GFP-tagged Qcr2. Importantly, mitochondrial respiration was dramatically impacted by the absence of Mrj1, as mutants demonstrated a reliance on the alternative oxidase for oxygen consumption and a lack of electron flow through complexes III and IV.

**FIG 11:**
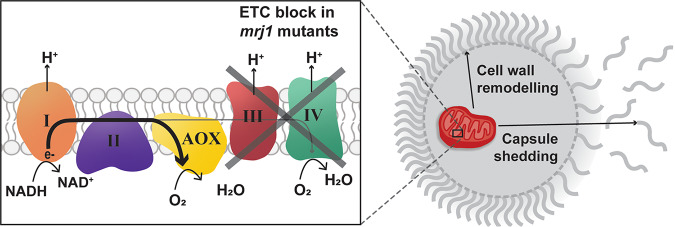
Model for the role of Mrj1 in mitochondrial function and virulence factor deployment. A cryptococcal cell is shown elaborating the polysaccharide capsule. The inset shows that in the *mrj1* knockout and J-domain-inactivated mutants, mitochondrial respiration is compromised. Specifically, we propose that the co-chaperone Mrj1 is required for electron flow through complexes III and IV, the proton motive force generated at these complexes, and the reduction of oxygen at complex IV. Rather, the alternative oxidase (AOX) is the site of oxygen reduction and termination of the electron transport chain (ETC). Furthermore, the mitochondrial defects in *mrj1* mutants are deemed to be responsible for the observed defect in cell wall architecture, increase in capsule shedding, and ultimately the reduced virulence of these mutants.

As mentioned, the growth of the *mrj1* mutants was affected differently by inhibitors of ETC complexes and the observed growth phenotypes were consistent with the changes in OCR when challenged with the same inhibitors. In particular, the OCR in the mutants was completely abolished upon treatment with the AOX inhibitor SHAM, and this corresponded to an inability to grow in the presence of SHAM. Inhibition of complex III had no effect on the OCR of mutants, again corresponding to no impact on growth in the presence of antimycin A or myxothiazol. Finally, addition of rotenone to inhibit complex I, which is upstream of AOX, impacted only the OCR of mutants in the absence of SHAM treatment. We interpret these findings as strong evidence that *mrj1* mutants are reliant on AOX for mitochondrial respiration. Overall, these data indicate that Mrj1 is required for completion of the ETC through complexes III and IV and that the alternative oxidase pathway is required for growth and respiration in the *mrj1* mutants, as illustrated in the model (Fig. 11).

The impact of Mrj1 on respiration was further supported by the physical interaction observed between Mrj1 and a core component of complex III, Qcr2. Complex III, also known as the cytochrome *bc*_1_ complex, is the site of the proton motive Q cycle (33, 38). In this process, electrons are transferred from ubiquinol to cytochrome *c*, and the electron transfer is coupled to the translocation of protons across the inner mitochondrial membrane. For each pair of electrons that enter the electron transport chain, four protons are pumped across the membrane at complex III, and another two protons are pumped across at complex IV (39). This in turn polarizes the mitochondria and creates a proton motive force which allows ATP to be generated (40). The explanation that loss of Mrj1 impairs the ETC at complexes III and IV is further supported by our analysis of mitochondrial membrane potential, as mutant strains would generate less proton motive force and less mitochondrial ROS in this situation. Consistent with this idea, the *mrj1* mutants had reduced fluorescence when stained with MitoTracker CMXRos, a membrane potential-dependent mitochondrial dye, and an increased proportion of cells with depolarized mitochondria as determined by JC-1 staining. Furthermore, this may explain the higher basal rates of OCR in the mutants as a compensation for generating less proton motive force per oxygen molecule consumed through the AOX protein. These findings are also consistent with the general growth defect and the lower optical density (OD) at stationary phase of *mrj1* mutants, which may be explained by decreased generation of proton motive force for ATP synthesis.

Our study adds to a growing body of evidence linking mitochondrial function to virulence in C. neoformans and the related Cryptococcus gattii species complex (37, 41–46). It is known, for example, that the variation in both intracellular proliferation rate within phagocytic cells and virulence among some genotypes of the C. gattii species complex can be attributed to differences in mitochondrial morphology (47, 48). Genetic studies also identified mitochondrial proteins with diverse functions that influence virulence in C. neoformans, and these proteins include Lys4 (amino acid biosynthesis), Vps45 (intracellular trafficking), Atm1 (mitochondrial iron uptake), Fzo1 (mitochondrial fusion), and Sod2 (superoxide dismutase) (41–45). Components of the ETC are also important for virulence as demonstrated by the reduced virulence of a mutant lacking alternative oxidase (46). More recently, a role for the ETC in capsule enlargement was established using inhibitors, including SHAM and antimycin A (37). Consistent with these findings, we also observed that antimycin A decreased capsule size, and we ruled out an off-target effect by showing that another complex III inhibitor, myxothiazol, also decreased capsule size. As previously suggested for other mutants which have reduced capsule size with increased capsule shedding, this phenotype is likely due to a defect in capsule attachment at the cell wall rather than synthesis of capsular polysaccharides (49–51). The *mrj1* mutants also had altered cell walls with less exposed chitin and chitosan, thus supporting the explanation that reduced capsule size was likely due to decreased capsule anchoring at the cell wall. Although the role of the ETC in the elaboration of virulence factors in C. neoformans has been interrogated using inhibitors, very little research has been done on the complexes directly. Our work and that of others on complex III inhibitors specific to fungi (52, 53) indicate that further analysis is warranted to study the biochemistry of this complex and other ETC components, as well as their assembly factors and chaperones, to better understand the fungal-specific differences and how they may be exploited to treat cryptococcosis. Promising recent work on this topic includes the finding that administration of the fungal-specific complex III inhibitor, ilicicolin H, reduces fungal burdens in a mouse model of disseminated cryptococcosis (53).

The contribution of mitochondria to virulence is an emerging area for C. neoformans and more broadly for fungal pathogens (54–56), and there is interest in mitochondria as targets for antifungal drug development (55–57). In our study, we found defects in the cell wall and capsule that we attributed to the defects in mitochondrial function in the *mrj1* mutants. Other proteins that contribute to mitochondrial function are known to mediate susceptibility to cell wall stress or capsule synthesis in C. neoformans. In particular, Mig1, Leu1, and Lys4 all influence cell wall-related phenotypes, and an observed capsule defect was attributed to cell wall changes in a *leu1* mutant (42, 58, 59). The connection between mitochondrial function and cell wall integrity is well established in C. albicans (60–65). For example, a screen of mutants hypersensitive to cell wall stress identified a role for the Ccr4 deadenylase in targeting transcripts for mitochondrial functions (62). This observation led to the subsequent identification of connections for specific mitochondrial proteins including Sam37 and Gem1 (52, 54). Sam37 is important for maintaining mitochondrial DNA (mtDNA), cell wall integrity, caspofungin tolerance, and ultimately virulence (61), while the mitochondrial GTPase, Gem1, plays a role in maintaining mitochondrial morphology and contributing to cell wall integrity in a Cek1-dependent manner (63). Recently, a role has been described for mitochondria in influencing the cell wall and contributing to a process called “masking” that results in avoidance of recognition by immune cells; the influence was attributed to modes of respiration or to hypoxia and connected to signaling via the cyclic AMP (cAMP)-protein kinase A (PKA) pathway (60, 64). Finally, it has been shown that ETC proteins including complex I subunits impact the expression of genes involved in cell wall integrity, particularly mannosylation functions (65). Although many of the mechanistic details connecting mitochondrial functions to cell wall synthesis and remodeling are not fully elucidated, the strong connection established in C. albicans provides a basis to continue investigation of such connections in other fungal pathogens. The roles we have discovered for Mrj1 in mitochondrial respiration, cell wall integrity, capsule attachment, and virulence fit with this emerging picture that mitochondrial function is a critical aspect of fungal pathogenesis.

Mrj1 is one of 24 proteins predicted to have J domains in C. neoformans and is distinct from any JDPs yet characterized in the model yeasts S. cerevisiae and S. pombe or in humans. There are several JDPs known to be in or associated with the mitochondria in S. cerevisiae, including Jac1 (iron-sulfur cluster biosynthesis), Pam18 (import of mitochondrial proteins), Mdj1 (protein folding in the mitochondria), Mdj2 (mitochondrial biogenesis), and Jid1 (function unknown) (20, 66). Mrj1, however, is a divergent JDP that lacks any similarity outside the highly conserved J domain with the JDPs in S. cerevisiae. In C. neoformans and other fungal pathogens, both the mitochondria (54, 56, 57, 59, 67) and the heat shock response (68–71) have been proposed as attractive targets for drug treatment and described as potential “Achilles’ heels.” Unlike other components of the heat shock response and mitochondria that are highly conserved between C. neoformans and humans, Mrj1 represents a promising target because it is divergent in amino acid sequence from any human proteins.

## MATERIALS AND METHODS

### Strains and media.

Cryptococcus neoformans var. *grubii* strain H99 (serotype A) was used in all experiments and as the background for mutant construction. All strains were routinely maintained on YPD medium (1% yeast extract, 2% peptone, 2% dextrose). Experiments to assess growth and other phenotypes were performed in yeast nitrogen base (YNB) medium with amino acids (BD Difco, Franklin Lakes, NJ) plus 0.5% dextrose, pH 5.6, unless otherwise specified. All strains in this study (see Table S3 in the supplemental material) were produced by biolistic transformation of linear constructs that were prepared using either three-step overlap PCR as previously described (72) or fast cloning (73). All constructs contained a resistance marker and were prepared with the primers, templates, and plasmids described in Table S3. The following constructs were made using overlap PCR; the *mrj1Δ* deletion construct was made using the primers Mrj1-1, -2, -3, -4, -5, and -6; the *MRJ1* complementation construct was made using the primers Mrj1c-1, -2, -3, -4, -5, and -6; the hemagglutinin (HA) tag construct was made using the primers Mrj1HA-1, -2, and -3, and Mrj1c-6; the Aox1-mCherry construct was made using the primers aox1mCh-1, -2, -3, -4, -5, and -6. The two green fluorescent protein (GFP)-tagged constructs were made using fast cloning and amplifying the vector using the primers ef1vF and ef1vR. The *MRJ1* insert was amplified using the primers ef1iF and ef1iR, whereas the *QCR2* insert was amplified using the primers Qcr2GFPiF and Qcr2GFPiR. Finally, the site-directed mutant was made first by using fast cloning to insert the full-length *MRJ1* complement construct (amplified with puc19mrj1F and puc19mrj1R) into the vector (amplified with puc19-1 and puc19-2), and then the primers SDMHQ1 and SDMHQ2 were used to mutate the codon (Table S3). All chemicals were obtained from Sigma (St. Louis, MO), unless otherwise specified.

10.1128/mBio.01127-20.10 TABLE S3 Strains, primers, and plasmids used in this study. Download Table S3, PDF file, 0.04 MB. Copyright © 2020 Horianopoulos et al. 2020 Horianopoulos et al. This content is distributed under the terms of the Creative Commons Attribution 4.0 International license.

### Mitochondrial localization.

A strain expressing a C-terminal fusion of GFP to Mrj1 was constructed in the background of the *mrj1Δ* mutant with transcription from the elongation factor 1 promoter at the genomic safe haven locus (29). The Mrj1-GFP-expressing cells were grown overnight in YNB, diluted to an optical density at 600 nm (OD_600_) of 1 and stained for 30 min with 50 nM MitoTracker CMXRos (Invitrogen, Carlsbad, CA) in YNB for colocalization studies.

For immunoblotting confirmation of Mrj1 localization, mitochondria were isolated using differential centrifugation as previously described (74) with a few modifications. Specifically, lysing enzymes from Trichoderma harzianum were used to generate spheroplasts, and glass beads were used for cell lysis. The integrity of the isolated mitochondria was examined using nonyl acridine orange and fluorescence microscopy. At the same time, total cell lysate was extracted using SEM buffer (0.25 M sucrose, 10 mM morpholinepropanesulfonic acid [MOPS] KOH [pH 7.2] and 1 mM EDTA) with 1% Triton X-100 and beating with glass beads. Protein concentrations were determined using Pierce BCA protein assay kit following the manufacturer’s instructions (Thermo Fisher Scientific, Waltham, MA), and 25 μg of lysate and mitochondrial protein was run in each well of a sodium dodecyl sulfate (SDS)-polyacrylamide gel before proceeding with immunoblotting. Proteins were transferred onto a polyvinylidene difluoride (PVDF) membrane (GE Healthcare, Boston, MA) using wet transfer at 70 V for 3 h. Membranes were blocked in Tris-buffered saline with Tween 20 (TBST) with 5% skim milk and incubated with the following antibodies at the indicated concentrations: monoclonal anti-HA (Thermo Fisher Scientific) at 1:10,000, anti-GFP labeled with horseradish peroxidase (HRP) (anti-GFP HRP) (Santa Cruz Biotechnology, Dallas, TX) at 1:750, anti-acetyl histone 3 at 1:5,000, and anti-mouse HRP (Bio-Rad, Hercules, CA) at 1:5,000, and anti-rabbit HRP (Bio-Rad) at 1:5,000. All immunoblots were visualized using chemiluminescence (GE Healthcare)

### Assessment of capsule size.

Capsule was induced using defined low-iron capsule-inducing medium (CIM) prepared as previously described (75). Briefly, cells were grown overnight in YPD and washed in low-iron water, and 10^6^ cells per ml were inoculated in CIM. Drug-treated cells were grown in the presence of 3 μg/ml antimycin A or 5 μM myxothiazol as indicated. Cells were imaged after 48 h of growth at 30°C with India ink staining using a Zeiss Plan-Apochromat 100×/1.46 oil lens on a Zeiss Axioplan 2 microscope. Images were obtained using an ORCA-Flash4.0 LT digital CMOS (complementary metal oxide semiconductor) camera (Hamamatsu, Hamamatsu City, Japan) and Zen software (Zeiss, Oberkochen, Germany). The capsule size was measured for 50 cells from each strain using ImageJ (76), and the difference in capsule size between strains was evaluated using a Kruskal-Wallis analysis of variance (ANOVA) in GraphPad Prism 6.0 (GraphPad Software, San Diego, CA).

### Assessment of capsule shedding.

The amount of shed capsule polysaccharide in the medium was assessed after 48 h of growth in CIM as previously described (77). Briefly, supernatant from each culture was diluted to an OD_600_ of 1, the supernatant was denatured at 70°C for 15 min, subjected to electrophoresis on an agarose gel, and blotted onto a nylon membrane (GE Healthcare). The membrane was incubated with a 1:1,000 dilution of the 18B7 monoclonal antibody, followed by incubation with a 1:5,000 dilution of anti-mouse HRP (Bio-Rad). Bound polysaccharide was visualized by chemiluminescence (GE Healthcare).

### Growth curves.

All growth curves were conducted in 96-well plates in a final volume of 200 μl inoculated with 1 × 10^5^ cells/ml. YNB with amino acids and 0.5% dextrose (BD Difco) at pH 5.6 was used as the base medium to test the sensitivities of mitochondrial stressors: 1.5 μg/ml rotenone, 3 mM salicylhydroxymate (SHAM), 3 μg/ml antimycin A (AA), 5 μM myxothiazol, or 15 mM KCN at 30°C. For iron utilization assays, iron was chelated using 150 mM bathophenanthrolinedisulfonic acid (BPS), and then the medium was supplemented with different iron sources, including 10 μM FeCl_3_, 10 μM FeSO_4_, or 10 μM hemin. Growth on alternative carbon sources was tested using YNB at pH 5.6 supplemented with 0.5% glycerol, lactate, or succinate. Thermotolerance was assessed by growing cells in YNB at either 30°C and 37°C.

### Virulence assays.

The WT, *mrj1Δ* mutant, and *mrj1Δ*::MRJ1 cells were grown in YPD overnight at 30°C, washed in phosphate-buffered saline (PBS), and resuspended at 1.0 × 10^6^ cells/ml in PBS. Ten female BALB/c mice aged 4 to 6 weeks old (Charles River Laboratories, ON, Canada) were inoculated with each strain by intranasal instillation with 50 μl of cell suspension (inoculum of 2 × 10^5^ cells per mouse). The mice were monitored daily postinoculation and euthanized by CO_2_ inhalation upon showing signs of morbidity. For the determination of fungal burdens in organs at endpoint, cardiac blood was retrieved, and organs were excised, weighed, and homogenized in 2 volumes of PBS using a MixerMill (Retsch, Haan, Germany). Serial dilutions of the homogenates were plated on YPD agar plates containing 50 μg/ml chloramphenicol, and CFU were counted after incubation for 48 h at 30°C. All experiments with mice were conducted in accordance with the guidelines of the Canadian Council on Animal Care and approved by the University of British Columbia’s Committee on Animal Care (protocol A17-0117). Significance in survival assays was determined using log rank tests, and significance in fungal burden was determined using Mann-Whitney U tests in GraphPad Prism 6.0.

### RNA extraction and quantitative real-time RT-PCR.

Overnight cultures were diluted 1 in 10 in fresh YPD and grown to log phase in a final volume of 25 ml for 6 h at 30°C with shaking. To study regulation at elevated temperatures, cells were collected, resuspended in prewarmed media, and grown with shaking for an additional 30 or 60 min as indicated. Cells were harvested, frozen in liquid nitrogen, and stored at −80°C. Cell pellets were lysed by bead beating, total RNA was extracted with an RNeasy kit (Qiagen, Hilden, Germany), and treated with Turbo DNase (Ambion, Austin, TX) according to the manufacturer’s recommendations. cDNA was synthesized using the Verso cDNA reverse transcription kit using oligo(dT) (Thermo Fisher Scientific). Quantitative reverse transcription-PCR (qPCR) was performed using Green-2-Go qPCR Mastermix and the primers listed in Table S3 (Bio Basic, Amherst, NY). The samples were run on an Applied Biosystems 7500 Fast real-time PCR system. Relative gene expression was quantified using the 2^−ΔΔCT^ method and normalized to *ACT1* and *GAPDH* expression (78). Statistical significance was evaluated using the unpaired *t* test.

### Flow cytometry.

Cells were grown for 16 h in YNB, diluted to an OD_600_ of 1, and stained for mitochondria and cell wall. Drug-treated cells were grown in the presence of 3 μg/ml antimycin A or 5 μM myxothiazol as indicated. For mitochondrial staining, cells were incubated with either 100 nM MitoTracker CMXRos in YNB, 250 nM nonyl acridine orange (NAO) in YNB, 5 μM JC-1 dye (Thermo Fisher Scientific) in PBS (pH 7.4), 2.5 μM MitoSOX Red Superoxide indicator (Thermo Fisher) for 30 min at 30°C with shaking at 150 rpm. After the cells were stained, the cells were washed three times in PBS to remove any extracellular dye. For cell wall staining, calcofluor white (CFW) and eosin Y were used to stain chitin and chitosan using previously reported concentrations and buffers (79). All flow cytometry data were collected on an Attune Nxt flow cytometer (Invitrogen). The following filters were used with their respective dyes: MitoTracker and MitoSox with YL1; NAO and eosin Y with BL1; JC-1 with BL1 and YL2; and CFW with VL1. Flow cytometry data were analyzed using FlowJo v10 software (FlowJo, LLC, Ashland, OR), and statistical significance was evaluated by performing ANOVAs with Dunn’s multiple comparisons in GraphPad Prism6 (GraphPad Software).

### Protein extraction.

An overnight culture was diluted 1 in 10 in fresh YPD and grown in a final volume of 50 ml for 6 h at 30°C with shaking and increased to 37°C for the last 30 min to induce expression of Mrj1 with an HA tag. Protein extracts were obtained as previously reported (80) with a modified lysis buffer (50 mM Tris-HCl [pH 7.5], 5 mM EDTA, 100 mM NaCl, 1% Triton X-100, and 1× EDTA-free protease inhibitor cocktail [Roche, Basel, Switzerland]), and water bath sonication of five 30-s cycles with 1 min in between cycles at 4°C using a Bioruptor Pico (Diagenode, Sparta, NJ). Protein concentration was determined using Pierce BCA protein assay kit following the manufacturer’s instructions (Thermo Fisher Scientific). Immunoblots were performed as previously described, and Ponceau S staining was performed on the membrane to assess equal loading and transfer.

### Co-immunoprecipitation and mass spectrometry (affinity purification and mass spectrometry [AP-MS]).

For immunoprecipitation, 1.5 mg of protein lysate was added to 25 μl of Pierce anti-HA magnetic beads (Thermo Fisher Scientific). Immunoprecipitation was performed according to the manufacturer’s instructions using a basic elution. Eluted proteins were either analyzed by immunoblotting or chloroform methanol precipitated for further processing and eventual MS analysis (81). Precipitated proteins were digested in RapiGest (Waters, Milford, MA) following the manufacturer’s in-solution digest protocol using 0.1% RapiGest. The solution containing the peptides was acidified to a pH of <2 using trifluoroacetic acid, and the RapiGest surfactant was precipitated out prior to purification using STop And Go Extraction (STAGE) tips (82). STAGE tipping was performed as previously described for acidic solutions in C18 medium using formic acid to acidify the solutions (83). The resulting peptides were resuspended in sample buffer containing 98% H_2_O, 2% acetonitrile, and 0.1% formic acid. All solvents for STAGE tipping were prepared using Optima liquid chromatography (LC)-MS quality reagents (Thermo Fisher Scientific).

Two microliters of each sample was subjected to reverse phase liquid chromatography for peptide separation using an RSLCnano Ultimate 3000 system (Thermo Fisher Scientific). Peptides were loaded on an Acclaim PepMap 100 precolumn (100 μm by 2 cm, C_18_, 3 μm, 100 Å; Thermo Fisher Scientific) with 0.07% trifluoroacetic acid at a flow rate of 20 μl/min for 3 min. Analytical separation of peptides was performed on an Acclaim PepMap rapid separation liquid chromatography (RSLC) column (75 μm by 50 cm, C_18_, 3 μm, 100 Å; Thermo Fisher Scientific) at a flow rate of 300 nl/min. The solvent composition was gradually changed within 94 min from 96% solvent A (0.1% formic acid) and 4% solvent B (80% acetonitrile, 0.1% formic acid) to 10% solvent B within 2 min, to 30% solvent B within the next 58 min, to 45% solvent B within the following 22 min, and to 90% solvent B within the last 12 min of the gradient. All solvents and acids were Optima grade for LC-MS (Thermo Fisher Scientific). Eluting peptides were on-line ionized by nano-electrospray (nESI) using the Nanospray Flex Ion Source (Thermo Fisher Scientific) at 1.5 kV (liquid junction) and transferred into a Q Exactive HF mass spectrometer (Thermo Fisher Scientific). Full scans in a mass range of 300 to 1,650 *m/z* were recorded at a resolution of 30,000 followed by data-dependent top 10 higher-energy collisional dissociation (HCD) fragmentation at a resolution of 15,000 (dynamic exclusion enabled). LC-MS method programming and data acquisition were performed with the XCalibur 4.0 software (Thermo Fisher Scientific).

MaxQuant 1.6.0.16 was used for protein identification and label-free quantification by searching MS/MS2 data against the Cryptococcus neoformans var. *grubii* H99 protein database (UP000010091, downloaded 19 October 2018). Default settings of MaxQuant were used with the addition of label-free quantification selected in group-specific parameters. The mass spectrometry proteomics data have been deposited in the PRIDE (84) partner repository with the data set identifier PXD013659. The results of the MaxQuant analysis were further processed and statistically analyzed using Perseus 1.6.0.7. Statistical significance of the enriched proteins in the tagged strain was evaluated using a one-sided *t* test with a false discovery rate (FDR) of 0.05 in Perseus.

### Seahorse oxygen consumption rate measurement.

The Seahorse XF cell mito stress test kit (Agilent, Santa Clara, CA) was used to measure the oxygen consumption rate (OCR) and to characterize mitochondrial respiration by extracellular flux analysis using an Agilent Seahorse XFe96 analyzer (Agilent). The Seahorse plate was coated with 0.01% poly-l-lysine, and 25,000 cells were allowed to adhere to each well from an overnight (16-h) culture in YNB (cell densities were tested and optimized prior to the assay). The cells were allowed to adhere for 30 min at 30°C before washing with fresh YNB. Additionally, 180 μl of Seahorse XF calibrant solution was added to each well of the Seahorse XF sensor cartridge to hydrate the XF utility plate. The hydrated cartridge was kept in a non-CO_2_ incubator at 30°C for 24 h, thereby removing CO_2_ from the media that would otherwise interfere with measurements. To allow the assay media to preequilibrate, 180 μl of YNB was added to each well, and the plate was placed in a 30°C in a non-CO_2_ incubator 1 h prior to the assay. Mitochondrial respiration was analyzed by sequential injections of modulators (titration of each modulator was performed prior to the experiment): SHAM (5 mM) used to inhibit the alternative oxidase; oligomycin (10 μM) used to block ATP synthase; carbonyl-cyanide-4-(trifluoromethoxy)phenyhydrazone (FCCP) (4 μM) used to activate uncoupling of the inner mitochondrial membrane, allowing maximum electron flux through the electron transport chain; and a mix of rotenone (4 μM) and antimycin A (4 μM) used together to inhibit complexes I and III, respectively. These drugs were also used in the absence of SHAM; however, rotenone and antimycin A were added separately to characterize the impact of complex I and complex III inhibition individually. These modulators were diluted in YNB and loaded into the injection ports of the hydrated sensor cartridge corresponding to the order of injection 1 h prior to the assay.
