# Supplementary material for: The Novel J-Domain Protein Mrj1 Is Required for Mitochondrial Respiration and Virulence in Cryptococcus neoformans
Source: mBio. 2020 Jun 9;11(3):e01127-20. doi: 10.1128/mBio.01127-20 (PMC7373193; doi:10.1128/mBio.01127-20)
Supplement: TABLE S1 [file mBio.01127-20-st001.pdf]

Supplemental Table S1. The gene IDs of the J domain proteins in *C. neoformans.*

C. neoformans gene ID	Predicted localization	*S. cerevisiae* ortholog	*S. pombe* ortholog
CNAG_00060	Nucleus/cytosol	Xdj1 (YLR090W)	Xdj1 (SPBC405.06)
CNAG_00233	Mitochondria	Pam18 (YLR008C)	Tim14 (SPAC824.06)
CNAG_00326	Cytosol	Djp1 (YIR004W)	Caj1/Djp1 type (SPAC4H3.01)
CNAG_00426	Nucleus/cytosol	Swa2 (YDR320C)	Ucp7 (SPAC17A5.12)
CNAG_00938	Mitochondria/ extracellular	-	-
CNAG_01347	Extracellular	-	-
CNAG_01696	Mitochondria	Mdj1 (YFL016C)	Mdj1 (SPCC4G3.14)
CNAG_01927	Mitochondria	-	-
CNAG_02038	Cytosol	-	DNAJC11 (SPCC63.03)
CNAG_02747	Plasma membrane	-	-
CNAG_02937	Cytosol	Sec63 (YOR254C)	Sec63 (SPBC36B7.03)
CNAG_03016	Nucleus	-	Spf31 (SPBC1734.05c)
CNAG_03487	Peroxisome	-	DNAJC9 (SPAC1071.09c)
CNAG_03944	Nucleus/cytosol	Ydj1 (YNL064C)	Mas5 (SPBC1734.11)
CNAG_04288	Mitochondria	Jac1 (YGL018C)	Jac1 (SPAC144.08)
CNAG_04820	Nucleus	-	Cwf23 (SPCC10H11.02)
CNAG_04976	Nucleus	Zuo1 (YGR285C)	Zuo1 (SPBC1778.01c)
CNAG_05252	Extracellular	Scj1 (YMR214W)	Scj1 (SPBC1347.05c)
CNAG_05538	Nucleus	Jjj1 (YNL227C)	Co-chaperone (SPAC6B12.08)
CNAG_05700	Plasma membrane	Erj5 (YFR041C)	Erj5 (SPAC2E1P5.03)
CNAG_06106	Nucleus/cytosol	Sis1 (YNL007C)	Psi1 (SPCC830.07c)
CNAG_06121	Mitochondria	-	DNAJ domain protein (SPCC63.13)
CNAG_06613	Plasma membrane	Hlj1 (YMR161W)	DNAJB12 (SPBC17A3.05c)
CNAG_07607	Nucleus	Caj1 (YER048C)	Caj1/Djp1 type (SPBC3E7.11c)

Proteins were identified by a BLAST analysis with the J domain consensus sequence (pfam00226) against the *C. neoformans* var. *grubii* H99 genome (taxid: 235443). For each protein, localization was predicted using WoLF PSORT, and the nearest orthologs in *S. cerevisiae* and *S. pombe* were determined through an ortholog search in FungiDB.
