# Supplementary figures and images for: The Novel J-Domain Protein Mrj1 Is Required for Mitochondrial Respiration and Virulence in Cryptococcus neoformans
Source: mBio. 2020 Jun 9;11(3):e01127-20. doi: 10.1128/mBio.01127-20 (PMC7373193; doi:10.1128/mBio.01127-20)

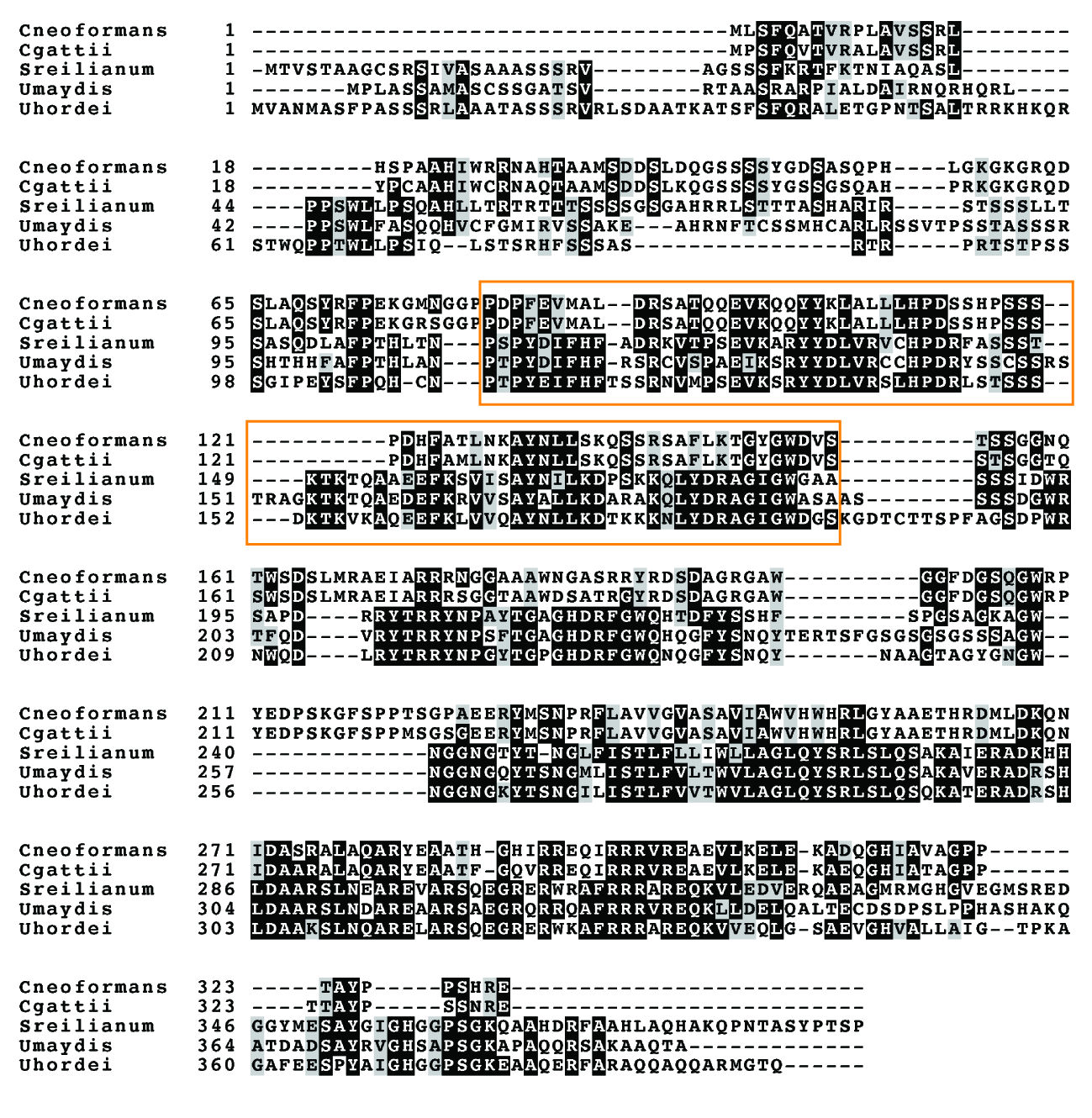

Supplement: FIG S1 [file mBio.01127-20-sf001.tif]

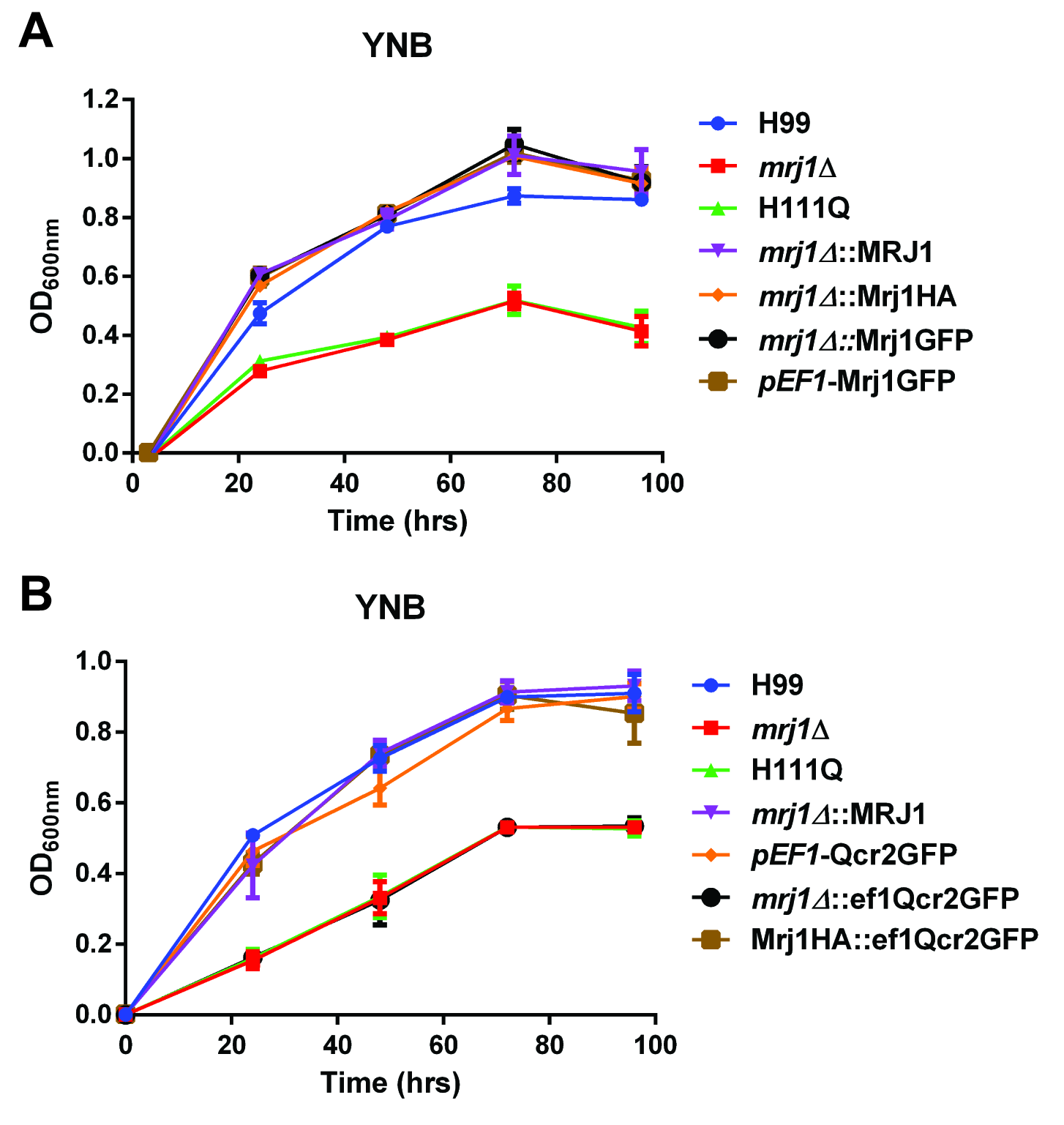

Supplement: FIG S2 [file mBio.01127-20-sf002.tif]

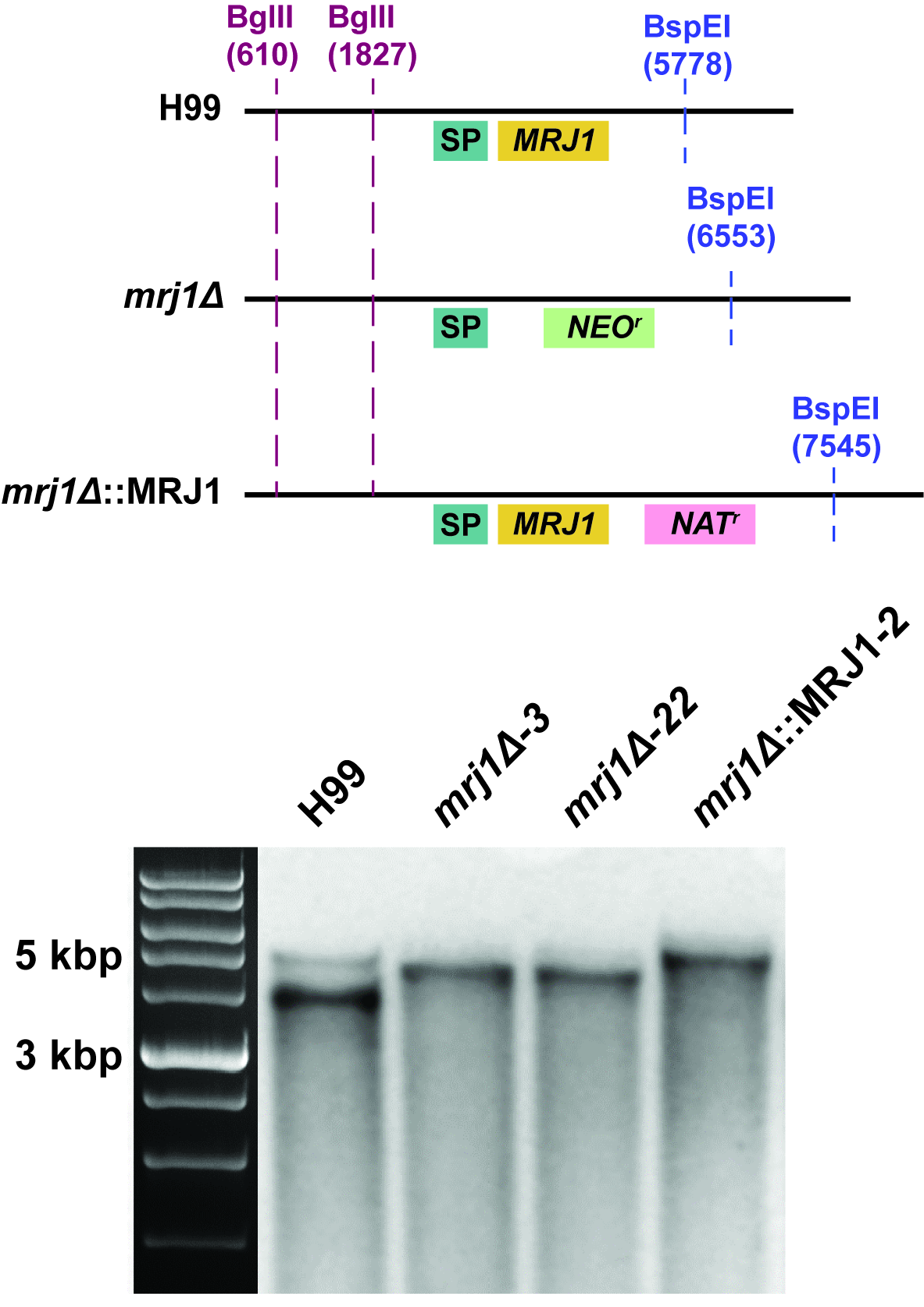

Supplement: FIG S3 [file mBio.01127-20-sf003.tif]

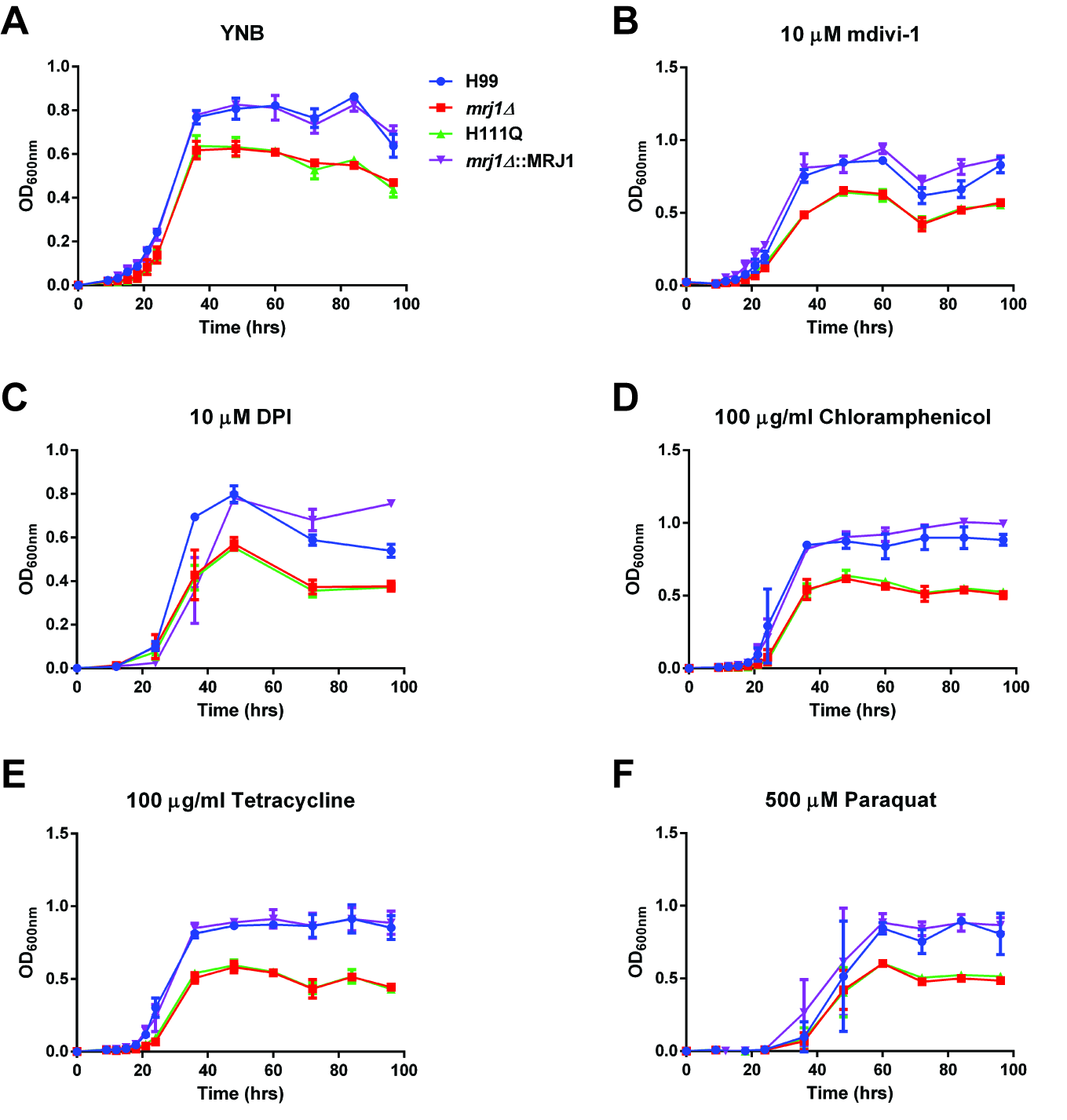

Supplement: FIG S4 [file mBio.01127-20-sf004.tif]
