# Supplementary material for: The Novel J-Domain Protein Mrj1 Is Required for Mitochondrial Respiration and Virulence in Cryptococcus neoformans
Source: mBio. 2020 Jun 9;11(3):e01127-20. doi: 10.1128/mBio.01127-20 (PMC7373193; doi:10.1128/mBio.01127-20)
Supplement: TABLE S2 [file mBio.01127-20-st002.pdf]

Supplemental Table S2. Proteins detected through AP-MS.

| Protein ID and description | Gene ID | Δlog2(LFQ intensity) | p value |
|---|---|---|---|
| J9VV75 Elongation factor Tu | CNAG_03263 | 2.328976 | 8.21E-05 |
| J9VVA3 40S ribosomal protein S27 | CNAG_03303 | 5.179834 | 0.000193 |
| J9VK51 40S ribosomal protein S21 | CNAG_01300 | 2.225234 | 0.000439 |
| J9VQ24 Eukaryotic translation initiation factor 5A | CNAG_01428 | 2.284287 | 0.001413 |
| J9VPD8 Ubiquinol-cytochrome c reductase core subunit 2 | CNAG_05179 | 0.547025 | 0.001514 |
| J9VXK4 Uncharacterized protein | CNAG_05556 | 0.551664 | 0.004263 |
| J9VPF5 Chaperone DnaK | CNAG_05199 | 0.53675 | 0.009141 |
| J9VU15 Chlorophyll synthesis pathway protein BchC | CNAG_01558 | 0.373762 | 0.021134 |
| J9VJ71 Large subunit ribosomal protein L8 | CNAG_05232 | 0.793948 | 0.022954 |
| J9W0B1 Large subunit ribosomal protein L4e | CNAG_04762 | 0.420177 | 0.027233 |
| J9VPT7 Small subunit ribosomal protein S28 | CNAG_06847 | 3.282871 | 0.044169 |
| T2BNZ0 40S ribosomal protein S6 | CNAG_01152 | 0.566963 | 0.049303 |
| J9VM66 Large subunit ribosomal protein L7e | CNAG_00656 | 0.239325 | 0.049357 |
| J9VD92 Glycerol-3-phosphate dehydrogenase [NAD(+)] | CNAG_00121 | 0.566241 | 0.051438 |
| J9VNN9 Large subunit acidic ribosomal protein P2 | CNAG_05762 | 0.175278 | 0.051835 |
| J9VY95 Serine hydroxymethyltransferase | CNAG_04601 | 1.746316 | 0.057449 |
| J9VHS2 ATP-dependent RNA helicase eIF4A | CNAG_00785 | 1.243365 | 0.061601 |
| J9VXH5 Small subunit ribosomal protein S18 | CNAG_04883 | 0.312419 | 0.062935 |
| J9VXN5 Malate dehydrogenase | CNAG_03225 | 0.720861 | 0.063672 |
| J9VQI0 Large subunit ribosomal protein L24e | CNAG_03283 | 0.362844 | 0.066223 |
| J9VXU0 Coatomer subunit beta | CNAG_03299 | 0.491694 | 0.071395 |
| J9VMD6 Uncharacterized protein | CNAG_07382 | 0.753946 | 0.071646 |
| J9VN56 Peptidyl-prolyl cis-trans isomerase | CNAG_03627 | 1.986648 | 0.075994 |
| J9VN50 NAD dependent epimerase/dehydratase | CNAG_02673 | 0.431397 | 0.078515 |
| J9VKV8 Large subunit ribosomal protein L10-like | CNAG_03739 | 1.15904 | 0.079671 |
| J9VVE7 Uncharacterized protein | CNAG_02129 | 0.162893 | 0.081175 |

| | | | |
|---|---|---|---|
| J9VMN2 Small subunit ribosomal protein S9 | CNAG_02331 | 0.33301 | 0.082397 |
| J9VGW8 Isocitrate dehydrogenase [NAD] subunit, mitochondrial | CNAG_07363 | 0.460527 | 0.093973 |
| J9W025 Uncharacterized protein | CNAG_06075 | 0.444824 | 0.093997 |
| J9VMD3 Large subunit ribosomal protein L5e | CNAG_02928 | 2.189067 | 0.094004 |
| J9VUB1 Histone H4 | CNAG_01648 | 0.453589 | 0.095566 |
| J9VGA5 Chaperonin GroES | CNAG_03892 | 1.95368 | 0.097768 |
| J9W2G5 Large subunit ribosomal protein L13e | CNAG_06095 | 0.296654 | 0.101937 |
| J9VQ69 Uncharacterized protein | CNAG_02843 | 0.821121 | 0.115016 |
| J9VT96 Large subunit ribosomal protein L18-A | CNAG_01224 | 0.756282 | 0.115986 |
| J9VUH9 Small subunit ribosomal protein S15 | CNAG_01679 | 1.042363 | 0.122922 |
| J9VSL1 Large subunit ribosomal protein L14e | CNAG_04799 | 0.307653 | 0.123153 |
| J9VS17 Protein BMH2 | CNAG_05235 | 0.420785 | 0.128391 |
| J9VRE8 DNA-binding protein | CNAG_00935 | 0.527351 | 0.129073 |
| J9VQI1 Ribosomal protein | CNAG_02144 | 0.512091 | 0.133167 |
| J9VMW1 Phosphoketolase | CNAG_02230 | 0.481277 | 0.139695 |
| J9VYP1 40S ribosomal protein S0RPS0 | CNAG_04114 | 0.147715 | 0.140988 |
| J9VLP5 40S ribosomalproteinS1RPS1 | CNAG_04004 | 0.214664 | 0.14123 |
| J9VKA9 Dihydrolipoyl dehydrogenase | CNAG_07004 | 0.71351 | 0.143395 |
| J9VDR3 Large subunit ribosomal protein L9e | CNAG_00034 | 0.224735 | 0.149683 |
| J9VXL7 40S ribosomal protein S8 | CNAG_03198 | 0.612696 | 0.151167 |
| J9VX38 Phosphatase | CNAG_01744 | 0.226343 | 0.152101 |
| J9VLE6 Polyubiquitin | CNAG_00370 | 0.53254 | 0.152741 |
| J9VJ21 Hsp60-like protein | CNAG_03891 | 0.317309 | 0.153552 |
| J9VK44 Obg-like ATPase1 | CNAG_02880 | 0.484435 | 0.154981 |
| O94746 FK506-binding protein1 FRR1 | CNAG_03682 | 0.911028 | 0.155079 |
| J9VKC4 GTP-binding protein ypt2 | CNAG_02817 | 0.757411 | 0.155564 |
| J9VZ70 Hsp71-likeprotein | CNAG_01727 | 0.197866 | 0.159568 |
| J9VH48 Oxidoreductase | CNAG_03983 | 0.486097 | 0.164255 |
| J9VEX3 Succinate-CoA ligase [ADP-forming] subunit beta, mitochondrial | CNAG_00747 | 0.217839 | 0.171253 |
| J9VY34 ADP, ATP carrier protein | CNAG_06101 | 0.13954 | 0.173064 |
| J9VPF2 Small subunit ribosomal protein S10e | CNAG_05814 | 0.140911 | 0.17479 |
| J9VI18 Fructose-bisphosphate aldolase 1 | CNAG_06770 | 1.042261 | 0.17691 |
| J9VLI9 Elongation factor 1-gamma | CNAG_00417 | 0.922928 | 0.178897 |

| | | | |
|---|---|---|---|
| J9VTA4 Large subunit ribosomal protein L12 | CNAG_01480 | 0.483133 | 0.183249 |
| J9VF13 Large subunit ribosomal protein L29 | CNAG_00771 | 0.281357 | 0.183948 |
| J9VS14 Uncharacterized protein | CNAG_04322 | 0.361668 | 0.186618 |
| J9VRH1 Glyceraldehyde-3-phosphate dehydrogenase | CNAG_06699 | 0.795795 | 0.193833 |
| J9VJQ8 Uncharacterized protein | CNAG_01446 | 1.296248 | 0.195803 |
| J9VM09 Inorganic pyrophosphatase | CNAG_02545 | 0.464104 | 0.195833 |
| J9VRA3 Nascent polypeptide-associated complex subunit alpha | CNAG_04985 | 0.355853 | 0.206547 |
| J9VQK9 UDP-glucuronate decarboxylase | CNAG_03322 | 0.517543 | 0.212004 |
| J9VP67 Ketol-acid reductoisomerase, mitochondrial | CNAG_05725 | 0.908009 | 0.215582 |
| J9VQ82 GTP-binding nuclear protein | CNAG_02257 | 0.177526 | 0.236306 |
| J9VXK6 Small subunit ribosomal protein S22-A | CNAG_01951 | 0.136144 | 0.236903 |
| J9W2T4 Heat shock 70kDa protein 4 | CNAG_06208 | 0.2464 | 0.237466 |
| J9VKK7 FK506-binding protein | CNAG_01148 | 0.412255 | 0.241999 |
| J9VWK6 Plasma membrane ATPase | CNAG_06400 | 0.122765 | 0.245607 |
| Q8TG24 Sulfate adenylyltransferase MET3 | CNAG_02202 | 0.406609 | 0.254424 |
| J9VFV3 Large subunit ribosomal protein L27Ae | CNAG_03747 | 0.416118 | 0.257884 |
| J9VES9 Large subunit ribosomal protein L27 | CNAG_00722 | 0.453715 | 0.260916 |
| J9VZS2 Small subunit ribosomal protein S5 | CNAG_01990 | 0.170432 | 0.260995 |
| J9VWJ8 F-type H-transporting ATPase subunit B | CNAG_01586 | 0.648847 | 0.26217 |
| J9VPH6 Cytochrome c oxidase subunit | CNAG_05839 | 0.583313 | 0.26828 |
| J9VX05 Large subunit ribosomal protein L7Ae | CNAG_05555 | 0.228957 | 0.271154 |
| J9VDW3 Uncharacterized protein | CNAG_00091 | 0.759155 | 0.273751 |
| J9VPE0 Small subunit ribosomal protein S23 | CNAG_03127 | 0.232122 | 0.276235 |
| J9VGS7 S-adenosylmethionine synthase | CNAG_00418 | 0.475967 | 0.277134 |
| J9VGR3 Proline-tRNA ligase | CNAG_04082 | 0.336247 | 0.277771 |
| J9VM00 Uncharacterized protein | CNAG_02332 | 0.152098 | 0.284362 |
| J9VXE8 Large subunit ribosomal protein L3 | CNAG_01884 | 0.51429 | 0.288272 |
| J9VSJ2 60S ribosomal protein L6 | CNAG_02234 | 0.096425 | 0.288305 |
| J9VP81 Nucleolar protein 58 | CNAG_05976 | 0.341036 | 0.294008 |

| | | | |
|---|---|---|---|
| J9VHC1 Large subunit ribosomal protein L28e | CNAG_04068 | 0.930017 | 0.296345 |
| J9VKM9 RuvB-like helicase | CNAG_00108 | 0.29914 | 0.302025 |
| J9VR33 60S ribosomal protein L36 | CNAG_03510 | 0.217086 | 0.311161 |
| J9VUT1 Uncharacterized protein | CNAG_03143 | 0.119304 | 0.317178 |
| J9VKK2 Small subunit ribosomal protein S13e | CNAG_01153 | 0.17138 | 0.319765 |
| J9VSC4 Large subunit ribosomal protein L21e | CNAG_02330 | 0.223204 | 0.320463 |
| J9VID8 40S ribosomal protein S12 | CNAG_02754 | 0.30991 | 0.320596 |
| J9VYC1 Mitochondrial-processing peptidase subunit beta | CNAG_03507 | 0.381299 | 0.320984 |
| J9VPP7 ATP synthase subunit beta | CNAG_05918 | 0.14197 | 0.327268 |
| J9VF80 Carbamoyl-phosphate synthase, large subunit | CNAG_07373 | 0.169629 | 0.328368 |
| J9VV89 Transaldolase | CNAG_01984 | 0.122715 | 0.329721 |
| J9VMZ2 UTP-glucose-1-phosphate uridylyltransferase | CNAG_02748 | 0.194761 | 0.343298 |
| T2BN71 Cytoplasmic protein, variant | CNAG_02943 | 0.174484 | 0.349277 |
| J9VW78 Nitric oxide dioxygenase | CNAG_01464 | 0.447664 | 0.353727 |
| T2BNJ3 Pyruvate kinase | CNAG_01820 | 0.447641 | 0.355376 |
| J9VQK7 Methylene tetrahydrofolate dehydrogenase (NADP) | CNAG_07746 | 0.283811 | 0.355761 |
| J9VUD6 Small subunit ribosomal protein S14 | CNAG_05904 | 0.384848 | 0.356247 |
| J9VFK3 NADH dehydrogenase (Quinone), G subunit | CNAG_03629 | 0.225204 | 0.359132 |
| J9VTE3 Small subunit ribosomal protein S17 | CNAG_01170 | 0.087453 | 0.367446 |
| J9VFY8 Small subunit ribosomal protein S16 | CNAG_03780 | 0.067509 | 0.369681 |
| J9VK98 Large subunit ribosomal protein L22e | CNAG_06811 | 0.086536 | 0.374094 |
| J9VUF9 Alcohol dehydrogenase (NADP) | CNAG_01896 | 0.071251 | 0.381268 |
| J9VP17 Pyruvate carboxylase | CNAG_05907 | 0.15668 | 0.384825 |
| J9W225 Large subunit ribosomal protein L23 | CNAG_01976 | 0.496819 | 0.390819 |
| J9VI11 6-phosphogluconate dehydrogenase, decarboxylating | CNAG_07561 | 0.090423 | 0.401699 |
| J9VZ02 Large subunit ribosomal protein L22 | CNAG_06447 | 0.1374 | 0.405479 |
| J9VL11 Tubulin alpha chain | CNAG_03787 | 0.090963 | 0.41976 |
| J9VR32 rRNA 2'-O-methyltransferase fibrillarin | CNAG_06919 | 0.066863 | 0.433809 |
| J9VVA4 Hsp90-like protein | CNAG_06150 | 0.045474 | 0.434473 |

| | | | |
|---|---|---|---|
| J9VKH0 Fructose-1,6-bisphosphatase I | CNAG_00057 | 0.119088 | 0.454058 |
| J9W0K1 ATP-citrate synthase | CNAG_04640 | 0.03753 | 0.454853 |
| J9VQB5 Nucleolar protein 56 | CNAG_02209 | 0.014821 | 0.475185 |
| J9VLX1 GTP-binding protein ypt3 | CNAG_02367 | 0.00986 | 0.4871 |
| J9VUU8 Small subunit ribosomal protein S25e | CNAG_02359 | 0.055796 | 0.489657 |
| J9VH03 Small subunit ribosomal protein S19e | CNAG_03000 | 0.001624 | 0.495987 |
| J9VTA9 Ribosomal protein L15 | CNAG_01486 | -0.0057 | 0.502997 |
| J9VTF4 Spermidine synthase | CNAG_03476 | -0.00231 | 0.508635 |
| J9VN13 Allergen | CNAG_06576 | -0.05765 | 0.513536 |
| J9VH15 Uncharacterized protein | CNAG_00534 | -0.04579 | 0.522417 |
| J9VU89 Small subunit ribosomal protein S20 | CNAG_01628 | -0.02702 | 0.528108 |
| J9W358 Solute carrier family 25 (Mitochondrial phosphate transporter), member 3 | CNAG_06377 | -0.00967 | 0.529122 |
| J9VSB6 Uncharacterized protein | CNAG_02340 | -0.02542 | 0.531953 |
| J9VHL5 Small subunit ribosomal protein S29 | CNAG_02811 | -0.17643 | 0.543972 |
| J9VIA4 C actin | CNAG_07323 | -0.04423 | 0.549513 |
| J9VKN9 Small subunit ribosomal protein S24e | CNAG_01332 | -0.02322 | 0.550173 |
| J9VRJ9 Adenosylhomocysteinase | CNAG_00886 | -0.03254 | 0.550428 |
| J9VQK8 Elongation factor 1-beta | CNAG_02714 | -0.23538 | 0.557613 |
| J9VTH3 Pyruvate decarboxylase | CNAG_04659 | -0.11719 | 0.56646 |
| J9VEY5 Mitochondrial carrier protein | CNAG_00512 | -0.03223 | 0.584233 |
| J9VJJ1 Large subunit acidic ribosomal protein P1 | CNAG_00655 | -0.39558 | 0.592237 |
| J9VEL7 Hsp75-like protein | CNAG_00334 | -0.05252 | 0.595219 |
| J9VHI6 Cytochrome c | CNAG_00716 | -0.12306 | 0.614464 |
| J9VTC1 60S ribosomal protein L20 | CNAG_04726 | -0.09151 | 0.620588 |
| J9VUR9 Polyadenylate-binding protein | CNAG_04441 | -0.12881 | 0.625463 |
| J9VIJ6 Aspartate-semialdehyde dehydrogenase | CNAG_00256 | -0.27209 | 0.652672 |
| J9VJJ6 Elongation factor 2 | CNAG_06840 | -0.08299 | 0.653724 |
| J9VVZ9 60S acidic ribosomal protein P0 | CNAG_03577 | -0.50572 | 0.66416 |
| J9VQN8 Phosphoglycerate kinase | CNAG_03358 | -0.21247 | 0.671785 |
| T2BP43 Chaperone activator, variant | CNAG_00305 | -0.17131 | 0.685217 |
| Q85SZ4 Cytochrome c oxidase subunit 2 COII | CNAG_09012 | -0.10885 | 0.689487 |
| J9VW24 Glucose-regulated protein | CNAG_06443 | -0.56355 | 0.70088 |
| J9VET5 Inosine-5'-monophosphate dehydrogenase | CNAG_00441 | -0.2712 | 0.711076 |
| J9VGV0 Enolase | CNAG_03072 | -0.23327 | 0.712103 |

| | | | |
|---|---|---|---|
| J9VL23 Transketolase | CNAG_07445 | -0.10617 | 0.716529 |
| J9W049 Uncharacterized protein | CNAG_06109 | -0.48822 | 0.724313 |
| J9VTL4 Alcohol dehydrogenase, propanol-preferring | CNAG_07745 | -0.33334 | 0.743418 |
| J9VMM1 Large subunit ribosomal protein L27e | CNAG_00779 | -0.28907 | 0.751742 |
| J9VZD5 Mannose-1-phosphate guanylyltransferase | CNAG_01813 | -0.29327 | 0.751814 |
| J9VLJ8 Chaperone regulator | CNAG_03944 | -0.11813 | 0.756085 |
| J9VQZ9 Elongation factor 3 | CNAG_01117 | -0.16018 | 0.757157 |
| J9VQS6 ATP synthase F1, delta subunit | CNAG_01204 | -0.54629 | 0.757325 |
| J9VTK1 Glutamate dehydrogenase | CNAG_01577 | -0.14534 | 0.758339 |
| J9VPP8 Uncharacterized protein | CNAG_03007 | -1.49327 | 0.761948 |
| J9VL03 F-type H-transporting ATPase subunit H | CNAG_00990 | -0.95938 | 0.766875 |
| J9VW13 Argonaute | CNAG_04609 | -0.41128 | 0.786994 |
| J9VLH1 Succinyl-CoA:3-ketoacid-coenzyme A transferase | CNAG_05031 | -0.47176 | 0.792457 |
| J9VP03 NADH dehydrogenase (Ubiquinone) Fe-S protein 3 | CNAG_07177 | -0.30602 | 0.798805 |
| J9VMP7 ATP-dependent RNA helicase ded1 | CNAG_00809 | -0.47034 | 0.803257 |
| J9W3X8 Guanine nucleotide-binding protein subunit beta-like protein | CNAG_05465 | -0.28767 | 0.805026 |
| J9VMQ3 40S ribosomal protein S30 | CNAG_00819 | -0.40132 | 0.821136 |
| J9VTW1 Uncharacterized protein | CNAG_01492 | -0.19519 | 0.828478 |
| J9VV35 Uncharacterized protein | CNAG_06113 | -1.0467 | 0.830603 |
| J9VU38 Glycerol-3-phosphate dehydrogenase [NAD(+)] | CNAG_01745 | -0.19873 | 0.847412 |
| J9VP88 ATP synthase subunit alpha | CNAG_05750 | -0.2225 | 0.853904 |
| J9VHE4 Small subunit ribosomal protein S11 | CNAG_00672 | -0.30886 | 0.863841 |
| J9VFA7 Tryptophan synthase | CNAG_00649 | -1.71774 | 0.871604 |
| J9VZI7 40S ribosomal protein S7 | CNAG_04445 | -0.1743 | 0.886767 |
| J9VD88 Small subunit ribosomal protein S3 | CNAG_00116 | -0.2012 | 0.897455 |
| J9VL89 Aconitate hydratase, mitochondrial | CNAG_01137 | -0.60179 | 0.90232 |
| J9W1R2 Tubulin beta chain | CNAG_01840 | -0.6707 | 0.921427 |
| J9W2J0 Elongation factor1-alpha | CNAG_06125 | -0.10612 | 0.930697 |
| J9VF99 40S ribosomal protein S4 | CNAG_00640 | -1.96764 | 0.932605 |
| J9VV50 Large subunit ribosomal protein L11 | CNAG_07839 | -0.21999 | 0.938843 |
| J9VQF1 Peroxiredoxin (Alkyl hydroperoxide reductase subunit C) | CNAG_03482 | -1.06991 | 0.9396 |

| | | | |
|---|---|---|---|
| J9VXF1 5-methyltetrahydropteroyltriglutamate-homocysteine S-methyltransferase | CNAG_01890 | -0.40916 | 0.941374 |
| J9VHP1 Voltage-dependent anion channel protein 2 | CNAG_02974 | -0.16227 | 0.944692 |
| J9VPA5 Large subunit ribosomal protein L24 | CNAG_04021 | -0.41265 | 0.956572 |
| J9VNW6 Small subunit ribosomal protein S15 | CNAG_06633 | -0.52989 | 0.969941 |
| J9VXQ3 THO complex subunit 4 | CNAG_03249 | -0.49186 | 0.971906 |
| J9VER4 Uncharacterized protein | CNAG_00410 | -0.71673 | 0.988179 |
| J9VFG4 Large subunit ribosomal protein L31e | CNAG_00703 | -0.67221 | 0.996642 |
| J9VN14 Nuclear GTP-binding protein | CNAG_02720 | -0.76756 | 0.996932 |
| J9VND3 Uncharacterized protein | CNAG_07665 | -0.70545 | 0.998369 |
| P48465 Actin | CNAG_00483 | -0.39028 | 0.99924 |

The Δlog2(LFQ intensity) is the average change in Label Free Quantification (LFQ) intensity from 3 replicates between the pull down with the Mrj1-HA tagged strain and the WT control. The p-value is derived from a one-sided t-test (FDR = 0.05) performed in Perseus. The mitochondrial proteins are highlighted.
