# Supplementary material for: The Novel J-Domain Protein Mrj1 Is Required for Mitochondrial Respiration and Virulence in Cryptococcus neoformans
Source: mBio. 2020 Jun 9;11(3):e01127-20. doi: 10.1128/mBio.01127-20 (PMC7373193; doi:10.1128/mBio.01127-20)
Supplement: TABLE S3 [file mBio.01127-20-st003.pdf]

Supplemental Table S3: Strains, primers, and plasmids used in this study.

| Strain | Genotype | Background | Primer name | Primer Sequence 5'-3' | Plasmids and templates |
|---|---|---|---|---|---|
| *mrj1Δ* | *mrj1::NEO* | H99 | Mrj1-1 | GACTCCCTCGTAGCCTTTCGGTTTAACG | H99 gDNA |
| | | | Mrj1-2 | GTCATCGTCTTACGGGGATTCTGCATCGCAGGCT GCGAGGATGTGAGCT | H99 gDNA |
| | | | Mrj1-3 | AGCTCACATCCTCGCAGCCTGCGATGCAGAATCC CCGTAAGACGATGAC | pJAF1 |
| | | | Mrj1-4 | TAGTTTCTACATCTCTTCTCCCCGCTCGTCGCTTTC ATACTCGTGT | pJAF1 |
| | | | Mrj1-5 | ACACGAGTATGAAAGCGACGAGCGGGGAGAAGAG ATGTAGAAACTA | H99 gDNA |
| | | | Mrj1-6 | TACACGACCAGCGAGCTTAATATCT | H99 gDNA |
| *mrj1Δ*::MRJ1 | *Mrj1::NAT* | *mrj1Δ* | Mrj1c-1 | GGGTGTCACTGCCAAGAGAA | H99 gDNA |
| | | | Mrj1c-2 | ACTGGCCGTCGTTTTACAACACGGATCAGACCCTA ACCGA | H99 gDNA |
| | | | Mrj1c-3 | TCGGTTAGGGTCTGATCCGTGTTGTAAAACGACGG CCAGT | pCH233 |
| | | | Mrj1c-4 | GAGTCATGTCGGCCTCAGAAGATGTAGAAACTAGC TTCCTGG | pCH233 |
| | | | Mrj1c-5 | CCAGGAAGCTAGTTTCTACATCTTCTGAGGCCGAC ATGACTC | H99 gDNA |
| | | | Mrj1c-6 | GCAGACTTGGGAAGCGTTTA | H99 gDNA |
| *mrj1Δ*::MRJ1 H111Q | *Mrj1H111Q::NAT* | *mrj1Δ* | pUC19-1 | CTGCAGGTCGACTCTAGAGG | pUC19 |
| | | | pUC19-2 | GCATGCAAGCTTGGCGTAATC | pUC19 |
| | | | pUC19mrj1F | TAGAGTCGACCTGCAGCTGCCAAGAGAATGAAGG TGGCTT | *mrj1Δ*::MRJ1 gDNA |
| | | | pUC19mrj1R | CGCCAAGCTTGCATGCGCGTTTATGCAGGAACCG AGTTATA | *mrj1Δ*::MRJ1 gDNA |
| | | | SDMHQ1 | CAAATTGGCTCTCCTGCTACAGCCCGATTCCTCCC ATC | pUC19mrj1 |

| | | | | | |
|---|---|---|---|---|---|
| | | | SDMHQ2 | GATGGGAGGAATCGGGcTGTAGCAGGAGAGCCAA TTTG | pUC19mrj1 |
| ef1-Mrj1-GFP | *pef1-Mrj1-GFP::HYG* | *mrj1Δ* | ef1vF | GTGAGCAAGGGCGAGGAGCT | pSDMA58 |
| | | | ef1vR | TTTGAAGTTTTCTGTGGAGATCGTT | pSDMA58 |
| | | | ef1iF | CACAGAAAACTTCAAAATGCTCTCCTTCCAAGCCA C | H99 gDNA |
| | | | ef1iR | TCGCCCTTGCTCACCTCCCGGTGCGAAGGAG | H99 gDNA |
| Mrj1-HA | *Mrj1-HA::NAT* | *mrj1Δ* | Mrj1HA-1 | GGGTGTCACTGCCAAGAGAATGAA | H99 gDNA |
| | | | Mrj1HA-2 | GTAATCAGGGACATCGTAAGGGTACTCCCGGTGC GAAGGAGGATAAGCTGT | H99 gDNA |
| | | | Mrj1HA-3 | CGATGTCCCTGATTACGCTTGACTGTCATTTGTAT GTATGCCAAATCTAGTGC | *mrj1Δ*::MRJ1 gDNA |
| | | | Mrj1c-6 | GCAGACTTGGGAAGCGTTTA | H99 gDNA |
| ef1-Qcr2-GFP | *pef1-Qcr2-GFP::HYG* | H99 | ef1vF | GTGAGCAAGGGCGAGGAGCT | pSDMA58 |
| | | | ef1vR | TTTGAAGTTTTCTGTGGAGATCGTT | pSDMA58 |
| | | | Qcr2GFPiF | CACAGAAAACTTCAAAATGTACTCCCTCAACAGGC TCC | H99 gDNA |
| | | | Qcr2GFPiR | TCGCCCTTGCTCACAAGACCGAGCTCGTCGCTAA | H99 gDNA |
| Mrj1-HA::Qcr2-GFP | *Mrj1-HA::NAT* *pef1-Qcr2-GFP::HYG* | Mrj1-HA | | See construct information for *Qcr2-GFP* | |
| ef1-Qcr2-GFP::Aox1-mCherry | *pef1-Qcr2-GFP::HYG* *Aox1-mCherry::NEO* | ef1-Qcr2-GFP | aox1mCH_1 | CTCAGCTTGTGCTGTGTTGC | H99 gDNA |
| | | | aox1mCh_2 | CTCGCCCTTGCTCACCTCAACGAGTCCTGAGCTTT TTTC | H99 gDNA |
| | | | aox1mCh_3 | CAGGACTCGTTGAGGTGAGCAAGGGCGAGGAG | pHD091 |
| | | | aox1mCh_4 | GTAAGAGAATGGGACGCCAGTGTGATGGATATCT GCAG | pHD091 |
| | | | aox1mCh_5 | TCCATCACACTGGCGTCCCATTCTCTTACTGCAAT CG | H99 gDNA |

| | | | | | |
|---|---|---|---|---|---|
| | | | aox1mCh_6 | GCTCTGGTGCATTGATGATAGC | H99 gDNA |
| Mrj1HA::ef1-Qcr2-GFP::Aox1-mCherry | *Mrj1-HA::NAT*<br>*pef1-Qcr2-GFP::HYG*<br>*Aox1-mCherry::NEO* | Mrj1-HA::Qcr2-GFP | | See construct information for *Aox1-mCherry* | |
| *mrj1Δ*::Qcr2-GFP | *mrj1::NEO*<br>*pef1-Qcr2-GFP::HYG* | *mrj1Δ* | | See construct information for *Qcr2-GFP* | |
| *mrj1Δ*::MRJ1::Qcr2-GFP | *Mrj1::NAT*<br>*pef1-Qcr2-GFP::HYG* | *mrj1Δ::*MRJ1 | | See construct information for *Qcr2-GFP* | |
| | | RT-qPCR primers | | | |
| | | | actinqF | CACCATTGGTAACGAGCGATTC | H99 cDNA |
| | | | actinqR | TGGTAGTACCACCAGACATGAC | H99 cDNA |
| | | | GAPDHqF | GCCGTAGGCAAGGTCATTC | H99 cDNA |
| | | | GAPDHqR | CCTTCAACTCAGGGCTCTC | H99 cDNA |
| | | | Mrj1qF | GCACAAGCACGTTACGAAG | H99 cDNA |
| | | | Mrj1qR | CGGTGCGAAGGAGGATAAG | H99 cDNA |
| | | | Ssa1qF | GCCAAGAACGGTCTTGAGTC | H99 cDNA |
| | | | Ssa1qR | TCCTTGGAAGCGGATTGC | H99 cDNA |
| | | | Erj5qF | CCCACACTGGTCAGACATAC | H99 cDNA |
| | | | Erj5qR | TTACCAGGCCCGGATTTC | H99 cDNA |

For each strain, the background strain that was transformed is indicated, and the primers are listed along with the plasmids and templates amplified to generate each construct. The primers used for RT-qPCR are also listed at the end of the table.
